# Bayesian Parameter Inference by Markov Chain Monte Carlo with Hybrid Fitness Measures: Theory and Test in Apoptosis Signal Transduction Network

**DOI:** 10.1371/journal.pone.0074178

**Published:** 2013-09-27

**Authors:** Yohei Murakami, Shoji Takada

**Affiliations:** Department of Biophysics, Division of Biology, Graduate School of Science, Kyoto University, Kyoto, Japan; University of Ulm, Germany

## Abstract

When exact values of model parameters in systems biology are not available from experiments, they need to be inferred so that the resulting simulation reproduces the experimentally known phenomena. For the purpose, Bayesian statistics with Markov chain Monte Carlo (MCMC) is a useful method. Biological experiments are often performed with cell population, and the results are represented by histograms. On another front, experiments sometimes indicate the existence of a specific bifurcation pattern. In this study, to deal with both type of such experimental results and information for parameter inference, we introduced functions to evaluate fitness to both type of experimental results, named quantitative and qualitative fitness measures respectively. We formulated Bayesian formula for those hybrid fitness measures (HFM), and implemented it to MCMC (MCMC-HFM). We tested MCMC-HFM first for a kinetic toy model with a positive feedback. Inferring kinetic parameters mainly related to the positive feedback, we found that MCMC-HFM reliably infer them with both qualitative and quantitative fitness measures. Then, we applied the MCMC-HFM to an apoptosis signal transduction network previously proposed. For kinetic parameters related to implicit positive feedbacks, which are important for bistability and irreversibility of the output, the MCMC-HFM reliably inferred these kinetic parameters. In particular, some kinetic parameters that have the experimental estimates were inferred without these data and the results were consistent with the experiments. Moreover, for some parameters, the mixed use of quantitative and qualitative fitness measures narrowed down the acceptable range of parameters. Taken together, our approach could reliably infer the kinetic parameters of the target systems.

## Introduction

In computational systems biology, mathematical models of gene regulatory networks or signal transduction networks are often represented by ordinary and partial differential equations. In these equations, there are kinetic parameters which characterize strengths of interactions or rates of biochemical reactions. However, all the values of kinetic parameters in the model are not always available from previous experiments and literatures. In these cases, unknown kinetic parameters need to be inferred so that the model simulation reproduces the known experimental phenomena. Parameter inference is very important for the mathematical modeling of biological phenomena, because it is known that network structures (network motifs) alone do not always determine the response or function of that network [1]. To infer unknown parameters, there are various methods used in systems biology [2]. Evolutionary strategy is one of the methods for parameter inference by iterative computation [3] and has already been used to estimate kinetic parameters of the mathematical models of metabolic pathway [4], circadian clock system of *Arabidopsis* [5] and mammal [6]. Simulated annealing [7] is an optimization algorithm and has already been used for parameter estimation of a biochemical pathway [8]. Although these methods are useful, they do not give us the information about credibility and uncertainty of unknown parameters with the distributions of unknown parameters.

In this respect, Bayesian statistics is a powerful method for parameter inference giving us the information about credibility and uncertainty of unknown parameters as a credible interval of posterior distribution. However, posterior distributions in Bayesian statistics are often difficult to obtain analytically. In these cases, Markov chain Monte Carlo methods (MCMC) [9], [10] can be used to obtain samples from posterior distributions. In conventional MCMC, explicit evaluation of a likelihood function is needed to evaluate a posterior distribution. Otherwise, when the likelihood function is analytically or computationally intractable, approximate Bayesian computation (ABC) [11] MCMC can be used. ABC-MCMC can evaluate posterior distribution without explicit evaluation of a likelihood function, but with simulation-based approximations in its algorithm [12]. ABC was implemented not only in MCMC but also in sequential Monte Carlo methods (SMC) [13], [14]. ABC-SMC has already been applied for parameter inference and model selection in systems biology [14]–[18].

Biological experiments are often performed with cell population, and the results are represented by histograms. For example, delay time and switching time of caspase activation after TRAIL treatment in apoptosis signal transduction pathway were represented by histograms [19]. Here, we call this kind of experimental result or data as a quantitative condition. On another front, experiments or observations sometimes indicate the existence of a specific bifurcation pattern. For example, experiments about RB-E2F pathway in cell cycle regulatory system and mitochondrial apoptosis signal transduction pathway indicate that those pathway work as bistable switches [20], [21]. Bistability indicates the existence of saddle-node bifurcation in mathematical modeling. Here, we call this kind of experimental result or data as a qualitative condition. In this study, to utilize those conditions for parameter inference, we introduce and call the functions which can evaluate the fitness to those conditions as quantitative and qualitative fitness measures respectively. Although conventional MCMC and ABC-MCMC evaluate posterior distribution with and without explicit evaluation of a likelihood function, respectively, none of these MCMC algorithms evaluate posterior distribution in the case that the experiments for parameter inference are a mixture of quantitative and qualitative conditions.

To overcome this problem, we formulated Bayesian formula for hybrid fitness measures (HFM) and implemented it to MCMC. We named the method MCMC-HFM which can deal with the mixture of qualitative and quantitative fitness measures. We first tested the MCMC-HFM to a kinetic toy model with a positive feedback. Starting with an assumed set of parameters that satisfies qualitative condition, we generated kinetic data with some noise. Using the generated data and qualitative condition, we tried to infer the kinetic parameters mainly related to the positive feedback. As the result, MCMC-HFM could reliably infer the kinetic parameters with use of both qualitative and quantitative fitness measures. Next, we applied the MCMC-HFM to a mathematical model of apoptosis signal transduction network, which was proposed before [22]. We tried to infer the kinetic parameters which are especially related to the implicit positive feedback because it is known to be important for characteristic system properties such as bistability and irreversibility of output [22]. As the result, MCMC-HFM could also reliably infer the kinetic parameters, especially those of which have experimental estimates without using these data and the results were consistent with experiments. We also examined 95% credible intervals of inferred parameter distributions, and tried to gain deeper understanding of the implicit positive feedback for bistability, irreversibility, and characteristic dynamics of output in the apoptosis model. This analysis allowed us to specify the important kinetic parameter and corresponding biochemical process for characteristic system properties. These results indicate that MCMC-HFM is a useful method for parameter inference and system analysis.

## Methods

We explain the derivation of MCMC-HFM algorithm. In Bayesian statistics, under given a likelihood function P(x_o_|θ) and a prior distribution π(θ), a posterior distribution π(θ| x_o_) is represented as follows:
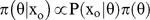



Here, θ is parameters and x_o_ is observed data. For computation of a posterior distribution, MCMC can be used and it generates samples from a posterior distribution. Conventional MCMC Metropolis-Hastings algorithm [9], [10] is as follows:

MCMC algorithm

MC1. Initialize θ_i_ i = 0.

MC2. Propose a candidate value θ*∼q(θ|θ_i_) where q is a proposal distribution.

MC3. Set θ_i+1_ = θ* with probability following α.
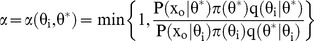
otherwise set θ_i+1_ = θ_i_.

MC4. If i<N, increment i = i+1 and go to MC2.

MCMC algorithm is designed as the stationary distribution is consistent with the target posterior distribution π(θ|x_o_). As shown above, conventional MCMC needs explicit evaluation of a likelihood function P(x_o_|θ) to judge whether a candidate value θ* is acceptable or not in step MC3. Conventional MCMC can be used in the case that the deviation between the experimental time series data and the simulated time series data is evaluated by probability distributions. For example, Eydgahi et al. used Gaussian distribution to evaluate the deviation [23]. They set the negative logarithm of the likelihood is correspondent to the chi-squared function which equals to the sum of squared differences between experimental data and simulated data at each time point. In contrast, ABC-MCMC evaluates a posterior distribution without explicit evaluation of a likelihood function, but with simulation-based approximations. Thus, ABC-MCMC is useful when the likelihood function is analytically or computationally intractable. ABC-MCMC algorithm is as follows:

ABC-MCMC algorithm

ABC1. Initialize θ_i_ i = 0.

ABC2. Propose a candidate value θ*∼q(θ|θ_i_) where q is a proposal distribution.

ABC3. Simulate a data set x*∼P(x|θ*).

ABC4. Set θ_i+1_ = θ* with probability following α.
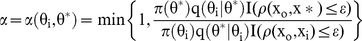
otherwise set θ_i+1_ = θ_i_.

ABC5. If i<N, increment i = i+1 and go to ABC2.

Here, I(C) is an indicator function which I(C) = 1 if a condition C is true, and 0 otherwise. ρ is a distance function and ε is a tolerance [12]. In ABC-MCMC algorithm, likelihood ratio P(x_o_|θ*)/P(x_o_|θ_i_) is approximated to I(ρ(x_o_, x*)≤ε))/I(ρ(x_o_, x_i_)≤ε)). Thus, likelihood ratio is coarsely approximated by 1 if simulated data and observed data are sufficiently “close”, and 0 otherwise [13]. ABC-MCMC can be used when an experimental data is a time series of protein concentration or gene expression level. The algorithm judges whether a simulated time series data with a model under θ is close enough to the experimental time series data or not. Toni et al. applied ABC-SMC algorithm for parameter inference of the repressilator model comparing time series data [14]. The idea and manner of ABC-MCMC, which judge the acceptance of parameters by all-or-none manner, might be intuitively applied when an experimental result support the existence of a specific phenomenon. In this case, unknown parameters are judged whether they reproduce the observed experimental phenomenon or not by all-or-none manner. An example is the case that an experimental result supports the existence of some specific bifurcation patterns such as saddle-node bifurcation or Hopf bifurcation in a mathematical model [24]–[27]. In this case, existence of a specific bifurcation pattern is judged by all-or-none manner. However, both MCMC and ABC-MCMC algorithms cannot, at least in these forms, deal with a mixture of quantitative and qualitative conditions. Thus, extensions and heuristic assumptions of these algorithms are needed to overcome this problem.

For this purpose, firstly, we consider the case that we have qualitatively different experiment data obtained in the same system. When we want to obtain a posterior distribution of parameters θ by n qualitatively different experiments, X_1_,…,X_n_, the posterior distribution are represented as follows:
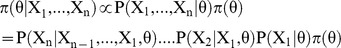



Here, n indicates the number of different experiments. Likelihood P(X_1_,…,X_n_|θ) can be decomposed into a multiplication of conditional probabilities. In this manner, we can utilize a number of different experimental data. In this study, we want to use both quantitative condition i.e. experimental data represented by histogram and qualitative condition i.e. experimental data which indicate the existence of specific bifurcation pattern. Then, we utilize the above idea, a multiplication of conditional probabilities, and employ an assumption to deal with both quantitative and qualitative conditions. A posterior distribution conditioned by a multiple quantitative and qualitative conditions is assumed as follows:
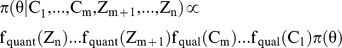



Here, we assumed and changed a likelihood term to a multiplication of a number of quantitative fitness measures (f_quant_) and qualitative fitness measures (f_qual_). In the equation, Z indicates a quantitative condition and C indicates qualitative condition. f_quant_(Z) is a quantitative fitness measure to a quantitative condition Z and f_qual_(C) is a qualitative fitness measure to a qualitative condition C. Rigorously speaking, fitness measures are not conditional probabilities. They are defined functions to evaluate the fitness of simulated data to experimental data. For a concrete evaluation of fitness, above equation is changed as follows:
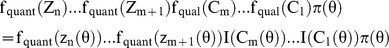



Here, quantitative fitness measures are changed to functions of z(θ). z is a concrete value calculated by numerical simulation under θ. Depending on a value of simulated z(θ), f_quant_ compare it to the experimental data represented by a histogram and returns a specific value. In this setting, we set f_quant_(z (θ)) is the value of “Frequency” at the corresponding class in a histogram of experimentally observed z. Quantitative fitness measures are changed to functions of condition C. Depending on a satisfaction of a condition C under parameters θ, f_qual_ returns a specific value. As a default, we assumed f_qual_(C) equals to I(C(θ)), indicator function. For example, when Hopf bifurcation is observed in numerical simulation under parameters θ, I(C(θ)) equals to 1, otherwise 0. In this example, a condition C equals to “existence of Hopf bifurcation”. We implemented this formula to the MCMC algorithm and then we could obtain MCMC-HFM algorithm as follows:

MCMC-HFM algorithm

HFM1. Initialize θ_i_ i = 0.

HFM2. Propose a candidate value θ*∼q(θ|θ_i_) where q is a proposal distribution.

HFM3. Simulate whether C_j_ (j = 1∼m) are satisfied or not under θ*.

HFM4. Set θ_i+1_ = θ* with probability following α.
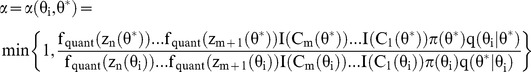



Otherwise set θ_i+1_ = θ_i_.

HFM5. If i<N, increment i = i+1 and go to HFM2.

MCMC-HFM algorithm is designed as the stationary distribution is consistent with the target distribution f_quant_(z_n_(θ))…f_quant_(z_m+1_(θ))I(C_m_(θ))…I(C_1_(θ))π(θ), with all qualitative conditions C_1_,…,C_m_ are satisfied. This was demonstrated in Text S1. By MCMC-HFM, we can deal with mixture of quantitative and qualitative fitness measures. Actually, about qualitative fitness measures, assumption of fitness measure may not be needed by setting a prior distribution to a distribution which satisfies qualitative conditions. However, for clarity of our idea and method, we introduced qualitative fitness measures.

## Results

### Flow of parameter inference

**Figure 1:**
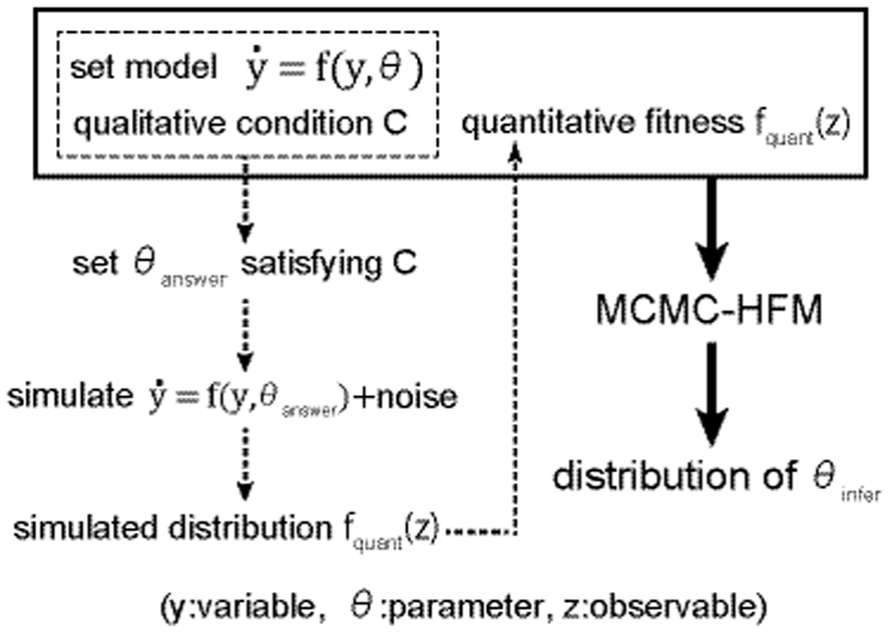
Flow chart of parameter inference. Flow chart of parameter inference. Dotted arrows and box with dotted line correspond to a preparation process of quantitative fitness f_quant_(z) specifically for the kinetic toy model. Bold arrows and box with bold line correspond to a general parameter inference process by MCMC-HFM.

In this section, we explain the flow of parameter inference (Figure 1) before detailed explanations. To show the efficiency of the use of hybrid fitness measures for parameter inference by MCMC-HFM, we applied the method to one test and one application. The test is done about a simple kinetic toy model and the application is done about an apoptosis signal transduction network.

In the test, we set the model and qualitative condition used for parameter inference by ourselves (box with dotted line in Figure 1). In addition, we prepared quantitative fitness measures f_quant_(z) by following procedures (dotted arrows in Figure 1). We set a vector of “true” parameters θ_answer_ satisfying qualitative condition. Then we generated distribution of observables by simulating the toy model with some noise into the model. We set the distribution of observables to f_quant_(z). Given the toy model, assumed qualitative conditions, and a simulated quantitative fitness (box with bold line in Figure 1), we performed MCMC-HFM and obtained distributions of parameters used for inference (bold arrows in Figure 1). The inferred parameters are compared with the “true” parameters.

In the application, we employed the apoptosis model of Legewie et al. 's [22]. Their model exhibits bistability and irreversibility, which we set as the qualitative conditions for parameter inference. As a quantitative fitness, we used the experimental data, instead of generation by simulations. Thus, in the application, we inferred distributions of parameters that fit with experiments by MCMC-HFM (flow from box with bold line to bold arrows).

### Test

#### Mathematical model of a kinetic toy model

**Figure 2:**
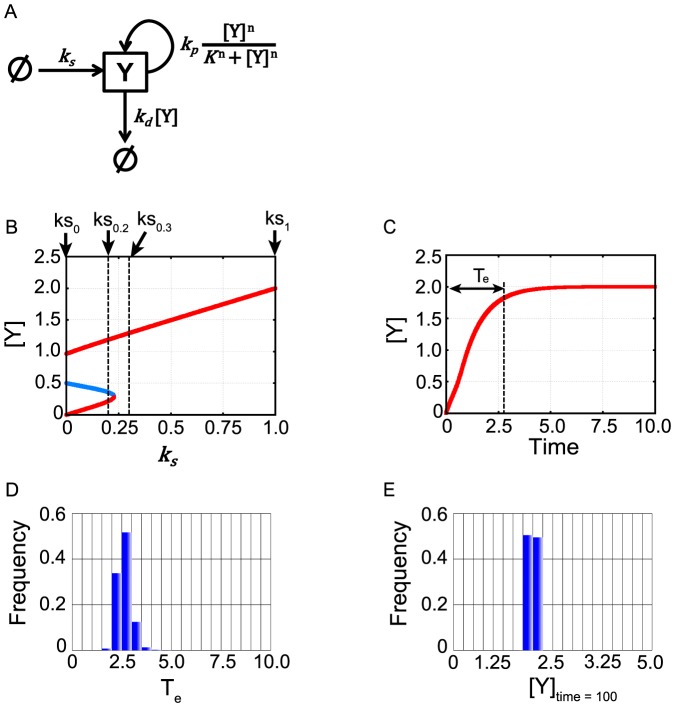
Mathematical model of the kinetic toy model. (A) Schematic diagram of the kinetic toy model. Y is a variable (protein). Arrows direct to Y represents production process. An arrow from Y represents degradation process. A lined circle represents a pool of Y. Equations correspond to the terms in the ordinary differential equation of the model. (B) Bifurcation diagram of the model. Red colored lines indicate stable steady states and the blue colored line indicates unstable steady state with “true” values of kinetic parameters θ_answer_. “ks_0_”, “ks_0.2_”, “ks_0.3_” and “ks_1_” indicate the k_s_ values (k_s_ = 0, 0.2, 0.3, 1.0 respectively) used as the conditions to infer kinetic parameters. (C) Time series of [Y]. T_e_ represents “execution time of Y production”. (D) Distribution of “execution time of Y production” abbreviated as “T_e_”. (E) Distribution of “concentration of Y at time = 100” abbreviated as “[Y]_time = 100_”.

As a test, we first applied MCMC-HFM to infer kinetic parameters of a simple kinetic toy model (Figure 2A). This system contains only one variable “Y” that corresponds to a protein. In the model, the protein is synthesized by zero-order reaction, and degraded by first-order reaction. In addition, the protein can enhance its own expression, forming a positive feedback represented by a Hill function. The ordinary differential equation of the model is
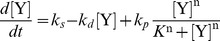



In the right hand side, the first term corresponds to synthesis of Y, the second term corresponds to degradation of Y, and the last term corresponds to the positive feedback. The synthesis rate constant, k_s_, is the input to the system. When other constants are set to the values θ_answer_ = (k_d_,k_p_,K) = (1.0,1,0,0.5), and the Hill coefficient is set to n = 5, the concentration of Y (represented as [Y]) shows bistability and irreversibility (Figure 2B). Here, θ_answer_ is the three dimensional vector of parameters. We note that we did not specifically define the unit of time, [Y] and parameters for simplicity. Those two features, bistability and irreversibility, were used as qualitative conditions for the parameter inference. In the application of MCMC-HFM to this model, we inferred the three constants, k_d_, k_p_ and K. Here, the Hill coefficient was fixed to n = 5 for simplification of the problem.

#### Generation of quantitative fitness measures in the kinetic toy model

To show the efficiency of the use of hybrid fitness measures, in addition to qualitative conditions i.e. bistability and irreversibility, we needed to prepare experimental results used as quantitative fitness measures. When the input is set to k_s_ = 1.0 over a whole time-series simulation, [Y] is produced and reaches to the almost saturated level by time  = 10 with θ_answer_ (Figure 2C). Based on this result, we decided to generate quantitative fitness measures i.e. histograms related to time series of Y production and amount of Y. In concrete terms, we decided to generate and use the data “execution time of Y production” and “concentration of Y at time  = 100” as quantitative fitness. The concrete definition of “execution time of Y” is that the time when [Y] reached the 90% of its maximum value in dynamics simulation or bifurcation analysis (“T_e_” in Figure 2C). The concrete definition of “concentration of Y at time  = 100” is that the value of [Y] at time  = 100 in dynamics simulation. To generate histograms of these two observables from the kinetic toy model with the “true” parameters values θ_answer_, we performed simulations for 10000 times by adding Gaussian noise into the model. When mean and variance of Gaussian noise was set to 0 and 1 respectively, execution time and concentration of Y at time  = 100 showed variation as shown in histograms in Figure 2D and E respectively. In this case study, we used these histograms as quantitative fitness measures for parameter inference. This setting and usage of histograms for parameter inference will be reasonable because experiments often performed in cell population and their results are sometimes shown by histograms [19].

#### Application of MCMC-HFM to the kinetic toy model

For parameter inference, we used four types of information, “bistability of Y” abbreviated as “B”, irreversibility of Y abbreviated as “I”, execution time of Y abbreviated as “T_e_” and “concentration of Y at time  = 100” abbreviated as “[Y]_time = 100_”. For the application of MCMC-HFM, “B” and “I” are qualitative conditions. In contrast, “T_e_” and “[Y]_time = 100_” are quantitative conditions. Therefore, we assigned “B” as C_1_, “I” as C_2_, “T_e_” as Z_3_, and “[Y]_time = 100_” as Z_4_ in MCMC-HFM algorithm (about the assignment to these symbols, see Methods section). Conditions C_1_ and C_2_ were judged by bifurcation analysis. The concrete definition of the condition C_1_ is that, there are two stable steady states and one unstable steady state when k_s_ = 0.2 (“ks_0.2_” in Figure 2B). There is one stable steady state when k_s_ = 0.3 (“ks_0.3_” in Figure 2B). There is one stable steady state when k_s_ = 1.0 (“ks_1_” in Figure 2B). When all of them are satisfied, f_qual_(C_1_) = I(C_1_(θ)) equals to 1, and 0 otherwise. Concrete definition of the condition C_2_ is that, in addition of the condition C_1_, there are two stable steady states and one unstable steady state when k_s_ = 0.0 (“ks_0_” in Figure 2B). When all of them are satisfied, f_qual_(C_2_) = I(C_2_(θ)) equals to 1, and 0 otherwise. In this case study, we used the histogram of “execution time of Y” (Figure 2D) as one quantitative fitness measure f_quant_(Z_3_) = f_quant_(z_3_(θ)), and the histogram of “concentration of Y at time = 100” (Figure 2E) as another quantitative fitness measure f_quant_(Z_4_) = f_quant_(z_4_(θ)). z_3_(θ) and z_4_(θ) were calculated by dynamics simulation under parameters θ. In this setting, f_quant_(z_3_(θ)) is the value of “Frequency” at the corresponding class of calculated z_3_(θ) in figure 2D, and f_quant_(z_4_(θ)) is the value of “Frequency” at the corresponding class of calculated z_4_(θ) in figure 2E. In bifurcation analysis, steady states concentrations of Y was calculated by solving the simultaneous equation obtained by setting the ordinary differential equation equals to zero with the standard Newton-Raphson method. Local stabilities of all the steady states were determined by evaluating eigenvalues of Jacobian matrices which were obtained by linearization of ordinary differential equation. In dynamics simulation, the ordinary differential equation was numerically solved by the fourth-order Runge-Kutta method with a time step of 0.01. Total calculation time was 100 (10000 steps). Initial concentration of Y in dynamics calculation was set to [Y]_time = 0_ = 0.

The prior distributions of parameters were set to follow the uniform distributions on a common logarithmic scale. Upper bound and lower bound were set to tenfold and one-tenth of the values of “true” parameter vector θ_answer_ respectively. Uniform distribution of kinetic parameters on a logarithmic scale has been used in robustness analysis in systems biology [28].

In the MCMC-HFM algorithm, the proposal distribution was set as the uniform distribution. Newly proposed parameter θ' is proposed by using unit random number “r” as follows.
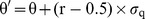



Here, θ is a vector consisting of common logarithm of kinetic parameters, and σ_q_ was set to 0.5 in this case study. Perturbation of kinetic parameter on a logarithmic scale has also been used in robustness analysis in systems biology [29].

In each step of MCMC-HFM, one of kinetic parameters was randomly chosen and perturbed by the proposal distribution q(θ'|θ). We performed totally 3.3×10^6^ Monte Carlo steps. The first 0.3×10^6^ steps were thrown away as the so-called burn-in period. Of the remaining 3.0×10^6^ steps, we recorded data every 3 steps, so that we collected totally 10^6^ data points for the parameter inference. From them, we can draw discrete marginal probability distributions as illustrated in Figure S1.

#### Parameter inference by posterior distribution for the kinetic toy model

To investigate the efficiency of the use of hybrid fitness, we conducted the parameter inference tests using different combination of fitness, comparing these results. First, we used only the both two types of the qualitative conditions, i.e., “BI”. Second, we used the two types of qualitative conditions and the one quantitative condition, “BIT_e_”. Third, we used “BI[Y]_time = 100_”. Lastly, we used all the four conditions, “BIT_e_[Y]_time = 100_”.

**Figure 3:**
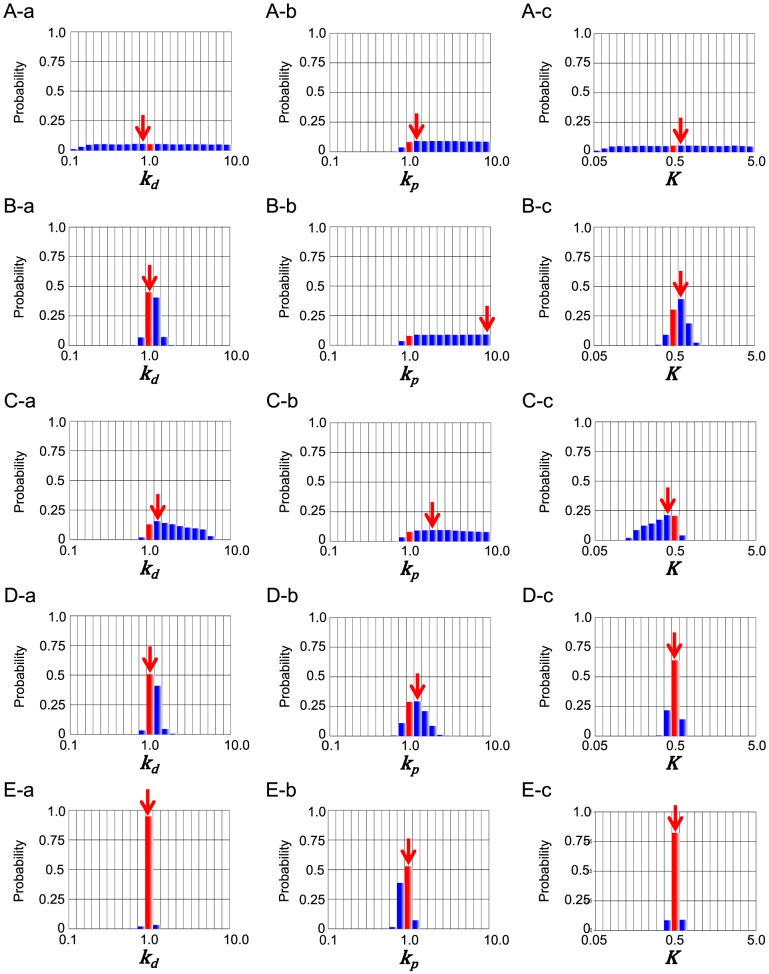
Marginal probability distributions of parameters in the kinetic toy model. Probability distributions with “BI” (A), those with “BIT_e_” (B), those with “BI[Y]_time = 100_” (C), those with “BIT_e_[Y]_time = 100_” (D) and those with ”BIT_e_[Y]_time = 100_” with weaker noise in quantitative fitness (E). Red bars represent the “true” values or parameters. Red arrows indicate the modes.

**Figure 4:**
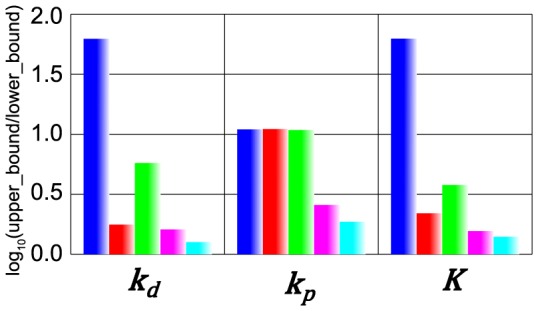
95% credible intervals of inferred parameters in the kinetic toy model. The 95% credible intervals are represented by common logarithm of the ratio of upper bound and lower bound of 95% credible intervals. Blue bars represent the case with “BI”. Red bars represent the case with “BIT_e_”. Green bars represent the case with “BI[Y]_time = 100_”. Magenta bars represent the case with “BIT_e_ [Y]_time = 100_”. Cyan bars represent the case with “BIT_e_ [Y]_time = 100_” with weaker noise in quantitative fitness.

In the present MCMC simulations, we define the representative parameter values of inference by the values at the mode, the peak of each marginal distribution (red arrows in Figure 3). We also calculated 95% credible intervals of each inferred parameter. Upper bound and lower bound of 95% credible interval were defined as external regions of bounds contain 2.5% data respectively. The common logarithm of the ratio of upper bound and lower bound of 95% credible interval was shown in Figure 4. A wider interval indicates lower credibility of the inferred value.

We first look into the parameter inference with “BI” (Figure 3.A). Figure 3.A showed that MCMC-HFM with “BI” well inferred (red arrow) the “true” values (the red bar) in all three parameters. However, the distributions of parameters were very wide which indicates the credibility of parameter inference is very low (Figure 4). In three parameters, k_p_ favored relatively larger values (Figure 3.A-b). This indicates that positive feedback needs to be strong to achieve bistability and irreversibility.

Next, we address the parameter inference with “BIT_e_” (Figure 3.B). MCMC-HFM well inferred the “true” values of k_d_ and K. The variability of k_d_ and K clearly decreased (Figure 3.B-a, B-c, Figure 4), which indicates the credibility of k_d_ and K inference get higher by the usage of quantitative condition, “T_e_”. However, MCMC-HFM could not well infer the “true” value of k_p_ and the credibility of k_p_ inference did not change (Figure 3.B-b, Figure 4). These results indicate that to reproduce the histogram of execution time in Figure 2.D, the degradation rate of Y (k_d_) and the working threshold of positive feedback (K) needed to be restricted, but not the strength of positive feedback (k_p_).

Next, we address the parameter inference with “BI[Y]_time = 100_” (Figure 3.C). MCMC-HFM well inferred the “true” values of k_d_ and K, and the variability of their distributions decreased, but not so much as “BIT_e_” did (Figure 3.C-a, C-c, Figure 4). k_p_ was still not so well inferred and had low credibility of inference (Figure 3.C-b, Figure 4). Thus, independent usage of quantitative condition, “T_e_” and “[Y]_time = 100_”, did not well infer k_p_.

However, when both of quantitative conditions were used with qualitative conditions “BI”, MCMC-HFM could infer all three parameters well (red arrows in Figure 3.D) with higher credibility (Figure 4) than independent usage of quantitative condition as shown in Figure 3.B and 3.C. This result may be interpreted as follows. The information “T_e_” restrict k_d_ and K as shown in Figure 3.B, then additional information “[Y]_time = 100_” could restrict k_p_. Thus, to infer the strength of positive feedback k_p_ with high credibility, the usage of both quantitative fitness measures is necessary.

If we changed the histograms in Figure 2.D and E to the histograms generated by adding much weaker Gaussian noise (variance was changed from 1 to 0.01) into the model, better inference with high credibility was accomplished (Figure 3.E, Figure 4).

Taken together, MCMC-HFM estimated the “true” values of kinetic parameters θ_answer_ very well with use of hybrid fitness measures. In addition, we could confirm that kinetic parameters were inferred to reproduce the histograms of “T_e_” and “[Y]_time = 100_” (Figure S2). Thus, MCMC-HFM could reliably infer the “true” values of kinetic parameters to reproduce the used histograms i.e. quantitative fitness measures for parameter inference. Generally, although the inferred range of parameter varies depending on the case, comparison of MCMC simulations in Figure 3 clarified that, with more types of fitness, we can narrow down acceptable range of parameters more. Thus, ability of hybrid use of quantitative and qualitative fitness measures by MCMC-HFM is indeed useful.

### Application

#### Mathematical model of apoptosis signal transduction network

**Figure 5:**
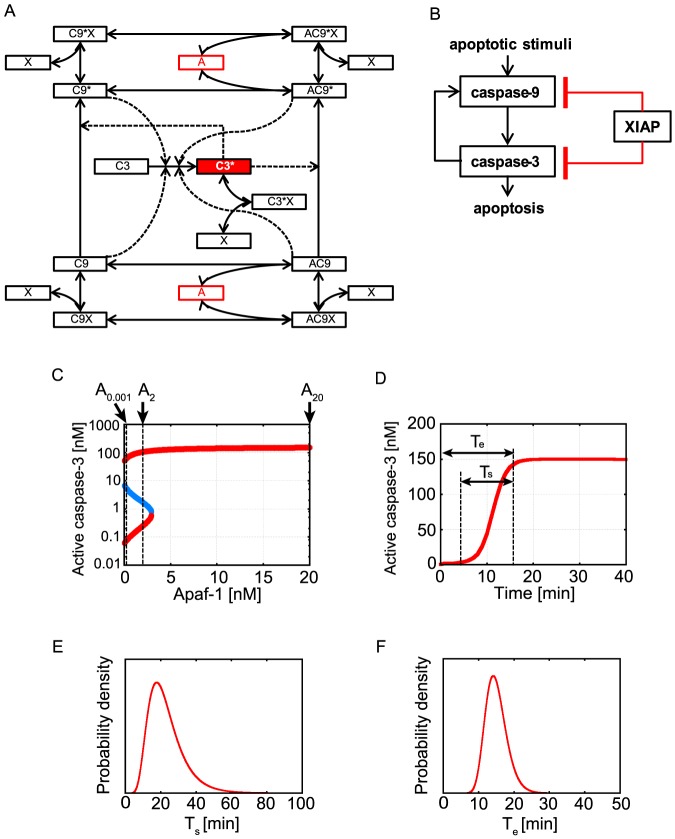
Mathematical model of the apoptosis signal transduction network. (A) Schematic diagram of the model. Solid arrows represent mass flows. Dotted arrows represent enhancement of the processes. One-way arrows between components represent irreversible processes. Two-way arrows between components represent reversible processes. Apaf-1 “A” is an input stimulus, and activated caspase-3 “C3*” is an output. Abbreviations are as follows: A: Apaf-1, C9: caspase-9, C3: caspse-3, X: XIAP. (B) Simplified diagram of the apoptosis signal transduction network at cytoplasm. Arrows represent activations. Lines with horizontal bar represent inhibition by binding and sequestering. Red colored interactions are implicit positive feedbacks. (C) Bifurcation diagram of the model. Red colored lines indicate stable steady states and the blue colored line indicates unstable steady state with the set of kinetic parameters used in Legewie et al's study. “A_0.001_”, “A_2_” and “A_20_” indicate the Apaf-1 concentrations (Apaf-1  =  0.001, 2.0, 20.0 respectively) used as the conditions to infer kinetic parameters. (D) Time series of active caspase-3 (C3*) with kinetic parameters used in Legewie et al's study. T_s_ represents “switching time of caspase-3 activation”. T_e_ represents “execution time of caspase-3 activation”. (E) Assumed function of switching time of caspase-3 activation. (F) Assumed function of execution time of caspase-3 activation.

As an application, we applied MCMC-HFM to infer kinetic parameters of the previously constructed Legewie et al. 's mathematical model of apoptosis signal transduction network (Figure 5.A, B) [22]. There are many mathematical models of apoptosis signal transduction network [19], [21], [28], [30]–[35] and related networks for cell fate decision [36]–[43]. In some of these models, bistability and irreversibility of output, such as caspase-3 which is an important enzyme for execution of apoptosis [44]–[46], are prominent characteristics of the system. Experimentally, it is known that activated caspase-3 level rises rapidly over a 10 ∼ 20 minutes period (here named “execution time” T_e_ in Figure 5.D) after mitochondrial outer membrane permeabilization (MOMP), which is the phenomenon that apoptotic signal is transmitted from mitochondria to cytoplasm [33]. In addition, the duration from the start to the finish of caspase-3 activation, named “switching time” T_s_ (Figure 5.D), varies among cell to cell, but is independent of the strength of death stimulus [19]. Legewie et al. 's model satisfies bistability and irreversibility of caspase-3 level (Figure 5.C), and caspase-3 activation occurs within 20 minutes (Figure 5.D) by using the kinetic parameters in their work [22]. However, several kinetic parameters in Legewie et al. 's model had not yet been determined experimentally. Thus, we decided to apply MCMC-HFM to their model.

In Legewie et al.'s model, input stimulus is Apaf-1 (represented as “A” in Figure 5.A) and output is active caspase-3 (represented as “C3*” in Figure 5.A). All biochemical reactions are represented by ordinary differential equations. In the application of MCMC-HFM to Legewie et al.'s model, we used the same ordinary differential equations described in their paper [22]. The model totally consists of 13 variables and 41 kinetic parameters. Legewie et al. revealed that so-called implicit positive feedback, which XIAP binds both caspase-3 and caspase-9 (Figure 5.B), plays an important role for bistability and irreversible activation of caspase-3. Thus, we tried to infer especially five kinetic parameters, association rate constant (k_asso_) between XIAP and five caspases which are directly related to implicit positive feedback. Here, dissociation rate constants of XIAP-caspase complexes and other kinetic parameters were fixed to the values in their paper [22] for simplification of the problem.

#### Application of MCMC-HFM to the apoptosis model

For parameter inference, we used four types of information, “bistability of caspase-3” abbreviated as “B”, “irreversible activation of caspase-3” abbreviated as “I”, “switching time of caspase-3 activation” abbreviated as “T_s_”, and “execution time of caspase-3 activation” abbreviated as “T_e_”. For the application of MCMC-HFM, “B” and “I” are qualitative conditions. On the other hand, “T_s_” and “T_e_” are quantitative conditions. Therefore, we assigned “B” as C_1_, “I” as C_2_, “T_s_” as Z_3_ and “T_e_” as Z_4_ in MCMC-HFM algorithm.

Conditions C_1_ and C_2_ were judged by bifurcation analysis. The concrete definition of the condition C_1_ is that, there are two stable steady states and one unstable steady state when Apaf-1 = 2.0 [nM], and active caspase-3 concentration of lower stable steady state is below 1.0 [nM] and higher stable steady state is over 1.0 [nM] (“A_2_” in Figure 5.C). There is one stable steady state when Apaf-1 = 20.0 [nM] and activated caspase-3 concentration is over 1.0 [nM] (“A_20_” in Figure 5.C). There are stable steady states when Apaf-1 = 0.001 [nM] and active caspase-3 concentration of lower stable steady state is below 1.0 [nM] (“A_0.001_” in Figure 5.C). The criterion of activated caspase-3 concentration 1.0 [nM] comes from the fact that this concentration is considered to be high enough for caspase-3 to cleave 10^6^–10^7^ molecules of cellular substrate within several hours when a cell volume is 1 picoliter [31], [33], [47]. When all of them are satisfied, f_qual_(C_1_) = I(C_1_(θ)) equals to 1, and 0 otherwise. Concrete definition of the condition C_2_ is that, in addition of the condition C_1_, there are two stable steady states and one unstable steady state when Apaf-1 = 0.001 [nM], and active caspase-3 concentration of lower stable steady state is below 1.0 [nM] and higher stable steady state is over 1.0 [nM] (“C_0.001_” in Figure 5.C). When all of them are satisfied, f_qual_(C_2_) = I(C_2_(θ)) equals to 1, and 0 otherwise.

Quantitative conditions, “switching time of caspase-3 activation” and “execution time of caspase-3 activation” were calculated by dynamics simulation. The concrete definition of “switching time of caspase-3 activation” is that the duration from the time when active caspase-3 concentration reached the 2.5% of its maximum value to the time when active caspase-3 concentration reached the 97.5% of its maximum value. The maximum value of active caspase-3 was chosen as the maximum concentration in each dynamics simulation and bifurcation analysis. The mean values of switching time in cell population are 19 to 27 minutes and standard deviations are 7.7 to 13 minutes, slightly differ depending on the strength of apoptotic stimulus, TRAIL concentration, as experimentally shown in Albeck's study [19]. The histogram of switching time was also shown in Albeck's study. However, they did not show the frequency of each class in the histogram explicitly. Thus, we needed to approximate the histogram of switching time. Because switching time is always larger than 0, log-normal distribution or gamma distribution can be used for approximation of the histogram. In this study, we adopted a log-normal distribution to mimic the switching time histogram. This is because it is known that skewed distributions often closely fit the log-normal distributions, and there are many examples across the sciences [48]. Assumed quantitative fitness measures of switching time is as follows:
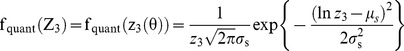



Here μ_s_ and σ_s_ were set as the expected value of z_3_ equals to 23 minutes and the standard deviation equals to 10 minutes (Figure 5.E). Numerically calculated switching time under parameters θ, z_3_(θ), is a variable of the quantitative fitness measure. The concrete definition of “execution time of caspase-3 activation” is that the time when active caspase-3 concentration reached the 90% of its maximum value in each dynamics simulation or bifurcation analysis. Experimentally, it is known that active caspase-3 level rises rapidly over a 10∼20 minutes period after MOMP [33]. However, there is no available histogram data of the distribution of execution time of caspase-3 activation. Thus, we adopted the log-normal distribution in the same way as the switching time. Assumed quantitative fitness measures of execution time is as follows:
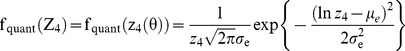



Here μ_e_ and σ_e_ were set as the expected value of z_4_ equals to 15 minutes and the standard deviation equals to 3 minutes, as most of parameter vectors reproduce 10∼20 minutes for execution time of caspase-3 (Figure 5.F) [33]. Numerically calculated execution time under parameters θ, z_4_(θ), is a variable of the quantitative fitness measure.

In bifurcation analysis, steady states concentrations of all proteins were calculated by solving the simultaneous equations obtained by setting all the ordinary differential equations equals to zero with the standard Newton-Raphson method. Local stabilities of all the steady states were determined by evaluating eigenvalues of Jacobian matrices which were obtained by linearization of ordinary differential equations. In dynamics calculation, the ordinary differential equations were numerically solved by the fourth-order Runge-Kutta method with a time step of 0.01. Total calculation time was 500 (50000 steps). Initial condition of the apoptosis model in dynamics calculation was shown in Table S1.

The prior distributions of parameters were set to follow the uniform distributions on a common logarithmic scale. Upper bound and lower bound were set to tenfold and one-tenth of the values used in Legewie et al.'s paper [22] respectively.

In MCMC-HFM algorithm, the proposal distribution was set as the uniform distribution. Newly proposed parameter θ' is proposed by using unit random number “r” as follows.
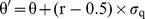



Here, θ is a vector consisting of common logarithm of kinetic parameters, and σ_q_ was set to 1.0 in this case study.

In each step of MCMC-HFM, one of kinetic parameters was randomly chosen and perturbed by the proposal distribution q(θ'|θ). We performed totally 5.5×10^6^ Monte Carlo steps. The first 0.5×10^6^ steps were thrown away as the so-called burn-in period. Of the remaining 5.0×10^6^ steps, we recorded data every 5 steps, so that we collected totally 10^6^ data points for the parameter inference. From them, we can draw discrete marginal probability distributions as illustrated in Figure S3.

#### Parameter inference by posterior distribution for the apoptosis model

To investigate roles of each type of fitness on parameters, we conducted the parameter inference using one to four types of fitness, comparing these results. First, we used only the first type of qualitative condition, i.e., “B”. Second, we used the two types of qualitative conditions, “BI”. Third, we used the two types of qualitative conditions and the one quantitative condition, “BIT_s_”. Fourth, we used “BIT_e_”. Lastly, we used all the four conditions, “BIT_s_T_e_”.

**Figure 6:**
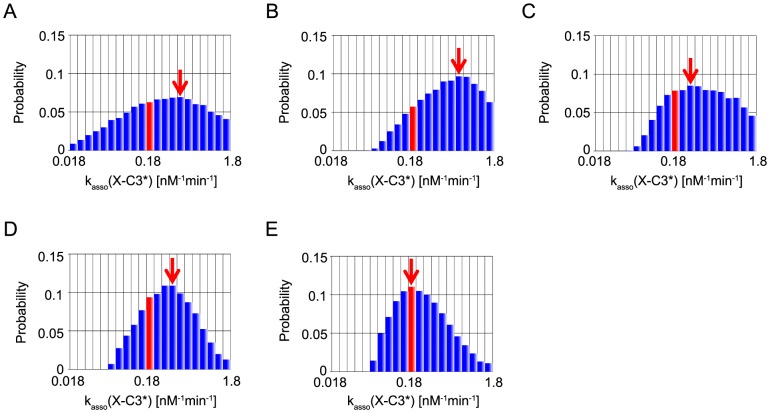
Marginal probability distributions of k_asso_ (X-C3*). Probability distribution with “B” (A), that with “BI” (B), that with “BIT_s_” (C), that with “BIT_e_” (D), and that with ”BIT_s_T_e_” (E). Red bars represent experimentally estimated values. Red arrows indicate the modes.

**Figure 7:**
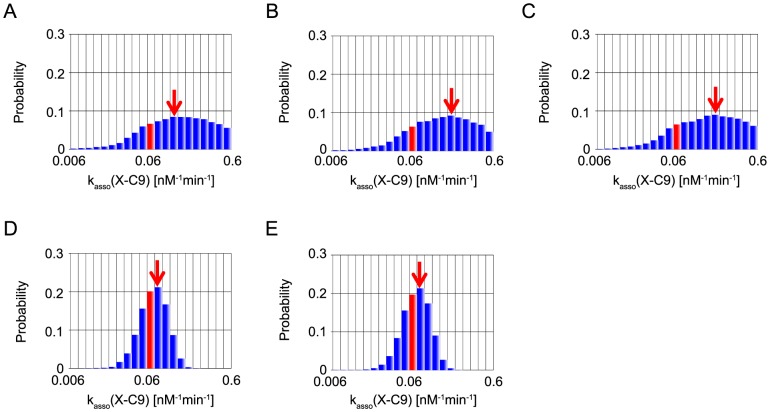
Marginal probability distributions of k_asso_ (X-C9). The same as captions in Figure 6.

**Figure 8:**
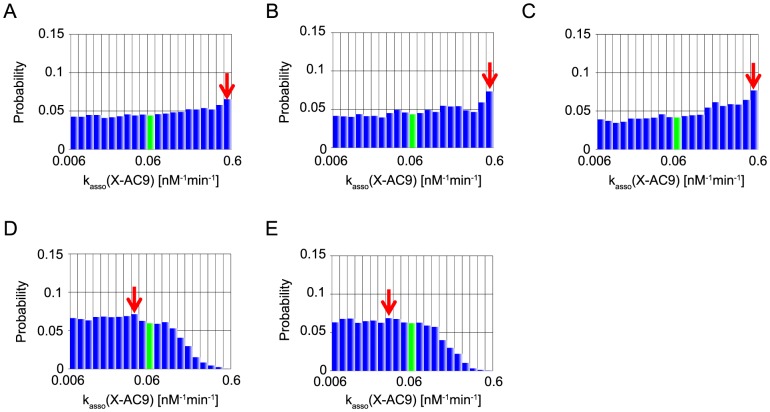
Marginal probability distributions of k_asso_ (X-AC9). The same as captions in Figure 6, except for green bars represent the used value in Legewie et al's study but not experimentally estimated.

**Figure 9:**
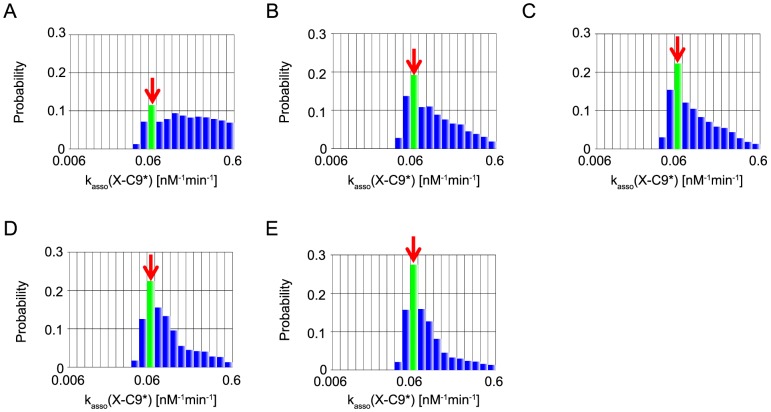
Marginal probability distributions of k_asso_ (X-C9*). The same as captions in Figure 8.

**Figure 10:**
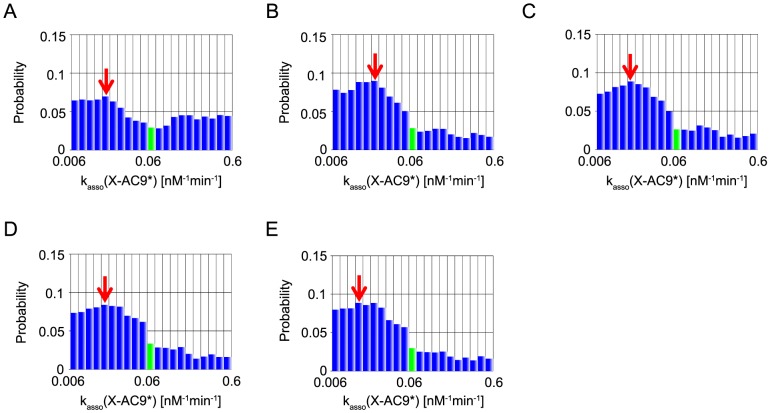
Marginal probability distributions of k_asso_ (X-AC9*). The same as captions in Figure 8.

In the present MCMC simulations, we also define the representative parameter values of inference by the values at the mode, the peak of each marginal distribution (red arrows in Figure 6–10). We note that, of the five parameters, two parameters, XIAP-C3* association rate constant (represented as k_asso_ (X-C3*)) and k_asso_ (X-C9) are experimentally characterized [49], [50] (values indicated by a red bar in Figure 6, 7), whereas the other three kinetic parameters have no experimental data and were assumed as the same values as that of k_asso_ (X-C9) in the reference [22] (values indicated by a green bar in Figure 8–10).

Specifically, we first look into the parameter inference of k_asso_ (X-C3*). Figure 6 A and B showed that MCMC-HFM with “B” or “BI” did not well infer (the red arrow) the experimentally characterized value (the red bar). On the other hand, MCMC-HFM with “BIT_s_” and “BIT_e_” (Figure 6.C D) inferred the value very close to the experimentally characterized value. Furthermore, MCMC-HFM with “BIT_s_T_e_” inferred the experimental value perfectly (Figure 6.E).

Next, we address the parameter inference of k_asso_ (X-C9). The inference with “B” or “BI” by MCMC-HFM (Figure 7.A, B) resulted in the parameter values somewhat deviated from the experimentally characterized value. The MCMC-HFM with “BIT_s_” also showed similar deviation (Figure 7.C). On the other hand, The MCMC-HFM with “BIT_e_” and with “BIT_s_T_e_” narrowed the parameter range and the mode (the red arrow) is very close to the experimental value (Figure 7.D, E). For both of the above parameter inference, clearly, the use of quantitative fitness together with qualitative fitness is powerful and thus MCMC-HFM provides a useful framework.

Next, as for k_asso_ (X-AC9), both of MCMC-HFM with “B”, “BI” and “BIT_s_” provided nearly uniform distribution without providing any information (Figure 8.A, B, C). On the other hand, MCMC-HFM simulations with “BIT_e_” and “BIT_s_T_e_” disfavored values larger than ∼0.06 although they still accept any values lower than ∼0.06. The mode was smaller than the value assumed in Legewie et al's paper (Figure 8.D, E) although the precise mode value does not seem robust in the current simulations due to the flatness of the distribution and the intrinsic error in simulations. We note again that the value of k_asso_ (X-AC9) was not determined experimentally and thus it is difficult to conclude if the inference was succeeded or not. At least, MCMC-HFM with “BIT_e_” and “BIT_s_T_e_” narrowed down the range of k_asso_ (X-AC9).

k_asso_ (X-C9*) were quite well inferred in all cases (Figure 9A–E). In all cases, the mode was consistent with the value assumed in Legewie et al's paper. Although the value of k_asso_ (X-C9*) was not determined experimentally, the inference by the current MCMC-HFM simulations strongly suggest that 0.06, the value used in the previous work, would be a good choice.

k_asso_ (X-AC9*) was inferred similarly by all the five simulations (Figure 10.A–E). Although the inferences are not strong, they all favor smaller rate constants than the value assumed in the work of Legewie et al. The fact that all five simulations gave similar results suggests that the existence of “B” alone disfavors, but not completely rules out, values larger than 0.06. Other types of fitness did not work for narrowing the parameter range.

Taken together, MCMC-HFM estimated experimentally estimated kinetic parameters, k_asso_ (X-C3*) and k_asso_ (X-C9), perfectly in consistent with experimental values.

#### Switching time and execution time for caspase-3 activation of inferred parameters

Next, we checked whether the switching time and execution time of caspse-3 activation are consistent with the assumed functions shown in Figure 5.E and F respectively.

**Figure 11:**
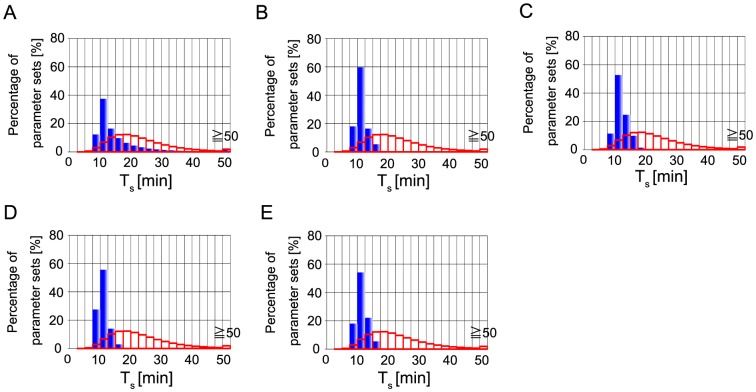
Calculated histograms of switching time of caspase-3 activation. Histogram of switching time of caspase-3 activation calculated with “B” (A), that with “BI” (B), that with “BIT_s_” (C), that with “BIT_e_” (D), and that with “BIT_s_T_e_” (E). Blue bars represent calculated results. Red outline box bars represent the approximated histogram of the function shown in Figure 5.E.

About the switching time of caspase-3 activation, T_s_, as shown in Figure 11.A, only with “B”, the inferred parameters showed log-normal like distribution, without quantitative condition, T_s_. However, the calculated histogram is not consistent with the approximated histogram of the assumed function (red outline box histogram in Figure 11). The peak of calculated distribution is located at faster position than the assumed function shown in Figure 5.E. By additional conditions ”I”, “T_s_” and “T_e_”, the distribution got sharper but the positions of peaks do not change (Figure 11.B–E). The calculated histograms are still not consistent with the approximated histogram of the assumed function. These results seem to indicate that other experimentally-unknown kinetic parameters in the model are not correct. This is discussed in Discussion section.

**Figure 12:**
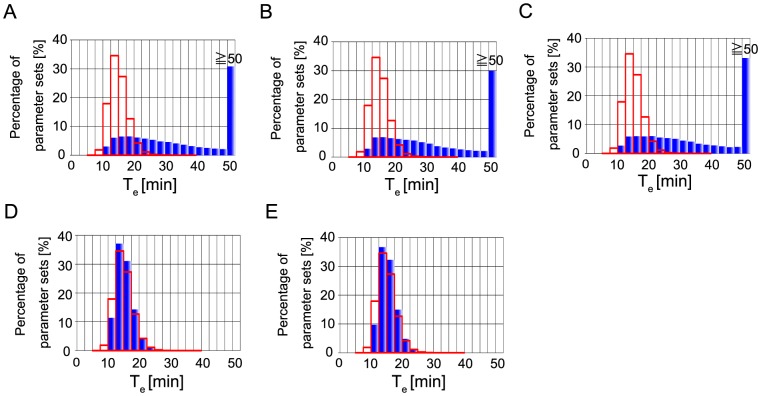
Calculated histograms of execution time of caspase-3 activation. Histograms of execution time of caspase-3 activation calculated with “B” (A), that with “BI” (B), that with “BIT_s_” (C), that with “BIT_e_” (D), and that with “BIT_s_T_e_” (E). Blue bars represent calculated results. Red outline box bars represent the approximated histogram of the function shown in Figure 5.F.

About the execution time of caspase-3 activation, T_e_, as shown in Figure 12.D and E, the inferred parameters showed clearly similar distribution to that in Figure 5.F, when “T_e_” was considered. Over 90% parameter sets showed 10∼20 minutes for caspase-3 activation. In the cases with “BI” and with “BIT_s_”, only about 20% parameter sets showed 10∼20 minutes for caspase-3 activation and calculated histograms are clearly far from the approximated histogram of the assumed function (Figure 12.A–C). Taken together, the inferred parameter sets could not well reproduce the function about “T_s_” assumed based on experimental results [19] as shown in Figure 11. On the other hand, the inferred parameter sets could reproduce the probability density function about “T_e_ “, assumed based on experimental results [33] as shown in Figure 12.

#### Credibility intervals for system analysis of the apoptosis model

**Figure 13:**
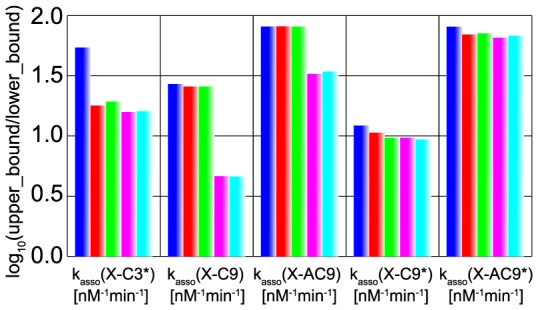
95% credible intervals of inferred parameters in the apoptosis model. The 95% credible intervals are represented by common logarithm of the ratio of upper bound and lower bound of 95% credible intervals. Blue bars represent the case with “B”. Red bars represent the case with “BI”. Green bars represent the case with “BIT_s_”. Magenta bars represent the case with “BIT_e_”. Cyan bars represent the case with “BIT_s_T_e_”.

Lastly, to quantify the acceptable range of parameters, we calculated 95% credible intervals of each inferred parameter. Upper bound and lower bound of 95% credible interval were defined as external regions of bounds contain 2.5% data respectively. Common logarithm of the ratio of upper bound and lower bound of 95% credible interval was shown in Figure 13. In Figure 13, k_asso_ (X-C3*), k_asso_ (X-C9) and k_asso_ (X-AC9) showed differences among the five MCMC simulations, while others did not show clear difference.

The 95% credible intervals of k_asso_ (X-C3*) became narrower by additional information of “I”. As seen in Figure 6, smaller values of k_asso_ (X-C3*) got unfavorable and higher values got favorable by additional information of irreversibility. This might be explained as follows. For irreversible activation of caspase-3, caspase-9 needs to activate caspase-3 constantly, and switch-on states of positive feedbacks needs to be sustained. For constant activation of caspase-3, a certain amount of caspase-9 has to be dissociated from XIAP. Thus, k_asso_ (X-C3*) tended to favor higher values to attract XIAP and 95% credible interval became narrower.

The 95% credible intervals of k_asso_ (X-AC9) and k_asso_ (X-C9) became narrower by additional information of “T_e_”. This result indicates that, free AC9 and, more dominantly, free C9 determine the timing of caspase-3 activation after MOMP (MOMP is at time  = 0 minutes in our simulation). This is actually consistent with our intuition. After Apaf-1 input, firstly, C9 and AC9 activate C3 to C3*. Then two positive feedbacks, one is the implicit positive feedback, and the other is the positive feedback that C3* activates C9 to C9* and AC9 to AC9* (Figure 5.A, B), are switched on and apoptotic stimulus is amplified. The switch-on timing of the positive feedbacks will determine the time when enough amount of caspase-3 is activated after Apaf-1 input, i.e. execution time of caspase-3 activation. Thus C9 and AC9 mainly determine the execution time of caspase-3 activation. Therefore, free C9 and free AC9 amounts need to be more strongly constrained, and thus the credible intervals became narrower.

In this manner, the current parameter inference process provides us lessons on which parameters are important for specific system properties. In addition, investigation about correlation coefficients and joint probability distributions between inferred parameters also provides us the relationships between parameters and specific system properties (See Text S2, Figure S4-S6 and Table S2).

In the two experimentally determined parameters, k_asso_ (X-C9) had narrow interval but k_asso_ (X-C3*) had still wide intervals. A wide credible interval of posterior distribution indicates that the information for parameter inference was not enough only with the information of bistability, irreversibility, switching time of caspase-3 activation and execution time of caspase-3 activation. As experimental results on the target system properties increases, the credible interval will become narrower and better inference will be accomplished.

## Discussions

In the present study, we introduced functions to evaluate fitness to experimental results, named fitness measures. Then we formulated Bayesian formula for hybrid fitness measures. We implemented it and developed MCMC-HFM algorithm to deal with a mixture of quantitative and qualitative fitness measures. We tested the MCMC-HFM algorithm for parameter inference in the kinetic toy model and the mathematical model of apoptosis signal transduction network. In the former, we inferred the kinetic parameters mainly related to positive feedback. As a result, MCMC-HFM could reliably infer the kinetic parameters with use of hybrid fitness measures. In the apoptosis model, we inferred the kinetic parameters which are related to the implicit positive feedback. As a result, MCMC-HFM could reliably infer the kinetic parameters, especially those of which values were experimentally estimated [49], [50]. Inferred parameter sets reproduced the function approximating the distribution of execution time of caspase-3. In the current study, the function was assumed based on the experimental result [33]. This function can be replaced with explicit experimental data represented as a histogram in possible future applications in the same way as the switching time of caspase-3 activation [19]. For inference, we define the representative parameter values of inference by the values at peak of each marginal distribution. This definition could reliably infer the kinetic parameters with use of hybrid fitness measures. Another definition of representative parameter values is the values at peak of joint distribution of all inferred parameters [23]. We used the uniform distribution as the proposal distribution in MCMC algorithms. Actually, almost the same results shown in Figure 3, 4, 6–13 and S1–5 were obtained when we used the normal distribution as the proposal distribution in MCMC (partly shown in Figure S7–14). These results indicate that MCMC-HFM is a useful and reliable method for parameter inference, and the results presented in the current study are reproducible.

In the apoptosis model, by the credible intervals of inferred parameters, joint probability distributions and correlation coefficients between inferred parameters, we could also specify the important relationships between kinetic parameters and corresponding biochemical processes, especially for irreversibility and execution time of caspase-3 activation. In the process of parameter inference by Bayesian statistics with MCMC, we can usually obtain many parameter sets, which can be used to understand and specify important biochemical processes in the target system as shown in the current study.

In the apoptosis model, inferred parameter sets reproduced well the assumed function of execution time of caspase-3 (Figure 5.F, Figure 12), but did not well reproduced the assumed function of the switching time of caspase-3 (Figure 5.E, Figure 11). This is not because of the restriction by two qualitative conditions, bistability and irreversibility. Because when we performed parameter inference only with a quantitative condition, “T_s_”, calculated histogram of T_s_ was not consistent with the assumed function (Figure S15). Thus, one possibility of inconsistency might be other experimentally-unknown kinetic parameters in the model are not correct. Legewie et al.'s model has a number of experimentally-unknown kinetic parameters [22]. In our case study, we fixed most of those kinetic parameters except for k_asso_ (X-AC9), k_asso_ (X-C9*) and k_asso_ (X-AC9*) in our parameter inference simulations. If we tried to infer all the unknown parameters in the model, the assumed function of the switching time of caspase-3 might be reproduced. Otherwise the mathematical model might need to be improved to be able to reproduce experimental results shown by Albeck et al. [19], [33]. We note that our calculation could not well reproduce the switching time of caspase-3 activation in the distribution level, but most of parameter sets showed acceptable switching time around ten and a few minutes compared with the experimental result (Figure 11).

Of the conditions used for parameter inference in the apoptosis model, “T_s_” did not largely narrow the distributions of any kinetic parameters (Figure 6–10) or strengthen correlation coefficients, which differ from other conditions (Table S2). This might indicate that other conditions have already had some information about the switching time of caspase-3. For example, we can easily see that “T_e_” has the information about “T_s_” because of its definition (Figure 5.D). This kind of interaction among a number of conditions, i.e. a number of experimental results, will often appear in future applications.

Robustness analysis of kinetic parameters in systems biology sometimes assumes the size of the parameter space as the measure of robustness. For example, the volume of the ellipsoid containing 95% of the parameters generated by Monte Carlo method was calculated and assumed as the measure of robustness [28]. In the same way, the 95% credible interval of posterior distribution obtained by parameter inference process by MCMC can be assumed to be the measure of robustness. For example, in the case study of the apoptosis model, k_asso_ (X-AC9) showed wide credible interval and roughly uniform distribution in the case with information “B” (Figure 8.A). This indicates the system is robust against perturbation of the strength of XIAP and AC9 association to maintain bistability of caspase-3. In contrast, the narrow 95% credible interval was k_asso_ (X-C9*) (Figure 9.A). This indicates the system is sensitive to perturbation of the strength of XIAP and C9* association to maintain bistability of caspase-3. In this manner, parameter inference by Bayesian statistics with MCMC can give us the information about the robustness of kinetic parameters. This point is also an advantage of parameter inference by Bayesian statistics with MCMC compared to other optimization algorithms which does not infer kinetic parameters as probability distributions.

In the same way as the MCMC-HFM algorithm, the idea to deal with mixture of quantitative and qualitative fitness measures simultaneously can be applied to SMC or so-called population Monte Carlo methods [51]. ABC-SMC has already been developed [13], [14] and applied to not only parameter inference but also model selection [14]–[18]. One of the problems of ABC-MCMC is that the efficiency of the algorithm is reduced when the ABC-MCMC sampler is trapped in an area of relatively low probability [13]. In the current study, the MCMC-HFM sampler was not strongly trapped in a low probability area as shown in trace plots in Figure S1 and S3. However, implementation to SMC or population Monte Carlo methods may improve the efficiency of sampling and may enable us to perform model selection more easily dealing with mixture of quantitative and qualitative fitness measures.

## Supporting Information

Figure S1 **Examples of trace plots of MCMC and probability distributions in the kinetic toy model.** (A) Trace plot and the probability distribution of k_p_ using “BI”. (B) Trace plot and the probability distribution of k_p_ using “BIT_e_[Y]_time = 100_”. Dotted lines indicate the 300000th step, of which left side is the burn in period.
(PDF)

Figure S2 **Calculated histograms of execution time of Y production “T_e_” and concentration of Y at time  = 100 “[Y]_time = 100_”.** Histograms of execution time of Y production, “T_e_”, and concentration of Y at time = 100, “[Y]_time = 100_” calculated with “BI” (A-a) and (A-b) respectively, those with “BIT_e_” (B-a) and (B-b) respectively, those with “BI[Y]_time = 100_” (C-a) and (C-b) respectively, those with “BIT_e_[Y]_time = 100_” (D-a) and (D-b) respectively, and those with “BIT_e_[Y]_time = 100_” with weaker noise in quantitative fitness (E-a) and (E-b). Blue bars represent calculated results. Red outline box bars represent the histogram generated by adding Gaussian noise into the model.
(PDF)

Figure S3 **Examples of trace plots of MCMC and probability distributions in the apoptosis model.** (A) Trace plot and the probability distribution of k_asso_ (X-C9) with “BI”. (B) Trace plot and the probability distribution of k_asso_ (X-C9) with “BIT_s_T_e_”. Dotted lines indicate the 500000th step, of which left side is the burn in period.
(PDF)

Figure S4 **Joint probability distributions of the pair of k_asso_ (X-C9) and k_asso_ (X-AC9).** Probability distribution with “B” (A), that with “BI” (B), that with “BIT_s_” (C), that with “BIT_e_” (D), that with “BIT_s_T_e_” (E).
(PDF)

Figure S5 Joint probability distributions of the pair of k_asso_ (X-C9*) and k_asso_ (X-C3*). Probability distribution with “B” (A), that with “BI” (B), that with “BIT_s_” (C), that with “BIT_e_” (D), that with “BIT_s_T_e_” (E).
(PDF)

Figure S6 **Simplified diagram of apoptosis signal transduction network focused on 4 implicit positive feedbacks.** Blue and numbered interactions represent implicit positive feedbacks. 1: C9-X-C3* implicit positive feedback, 2: AC9-X-C3* implicit positive feedback, 3: C9*-X-C3* implicit positive feedback, 4: AC9*-X-C3* implicit positive feedback.
(PDF)

Figure S7 **Marginal probability distributions of parameters in the kinetic toy model (proposal distribution in MCMC set to normal distribution).** Probability distributions with “BI” (A), those with “BIT_e_” (B), those with “BI[Y]_time = 100_” (C), those with “BIT_e_[Y]_time = 100_” (D), and those with ”BIT_e_[Y]_time = 100_” with weaker noise in quantitative fitness (see main text) (E). Red bars represent the “true” values of parameters. Red arrows indicate the modes.
(PDF)

Figure S8 **95% credible intervals of inferred parameters in the kinetic toy model (proposal distribution in MCMC set to normal distribution).** The 95% credible intervals are represented by common logarithm of the ratio of upper bound and lower bound of 95% credible intervals. Blue bars represent the case with “BI”. Red bars represent the case with “BIT_e_”. Green bars represent the case with “BI[Y]_time = 100_”. Magenta bars represent the case with “BIT_e_ [Y]_time = 100_”. Cyan bars represent the case with “BIT_e_ [Y]_time = 100_” with weaker noise in quantitative fitness.
(PDF)

Figure S9 Marginal probability distributions of k_asso_ (X-C3*) (proposal distribution in MCMC set to normal distribution). Probability distribution with “B” (A), that with “BI” (B), that with “BIT_s_” (C), that with “BIT_e_” (D), and that with ”BIT_s_T_e_” (E). Red bars represent experimentally estimated values. Red arrows indicate the modes.
(PDF)

Figure S10 **Marginal probability distributions of k_asso_ (X-C9) (proposal distribution in MCMC set to normal distribution).** Probability distribution with “B” (A), that with “BI” (B), that with “BIT_s_” (C), that with “BIT_e_” (D), and that with ”BIT_s_T_e_” (E). Red bars represent experimentally estimated values. Red arrows indicate the modes.
(PDF)

Figure S11 **Marginal probability distributions of k_asso_ (X-AC9) (proposal distribution in MCMC set to normal distribution).** Probability distribution with “B” (A), that with “BI” (B), that with “BIT_s_” (C), that with “BIT_e_” (D), and that with ”BIT_s_T_e_” (E). Green bars represent the used value in Legewie et al's study but not experimentally estimated. Red arrows indicate the modes.
(PDF)

Figure S12 Marginal probability distributions of k_asso_ (X-C9*) (proposal distribution in MCMC set to normal distribution). Probability distribution with “B” (A), that with “BI” (B), that with “BIT_s_” (C), that with “BIT_e_” (D), and that with ”BIT_s_T_e_” (E). Green bars represent the used value in Legewie et al's study but not experimentally estimated. Red arrows indicate the modes.
(PDF)

Figure S13 Marginal probability distributions of k_asso_ (X-AC9*) (proposal distribution in MCMC set to normal distribution). Probability distribution with “B” (A), that with “BI” (B), that with “BIT_s_” (C), that with “BIT_e_” (D), and that with ”BIT_s_T_e_” (E). Green bars represent the used value in Legewie et al's study but not experimentally estimated. Red arrows indicate the modes.
(PDF)

Figure S14 **95% credible intervals of inferred parameters in the apoptosis model (proposal distribution in MCMC set to normal distribution).** The 95% credible intervals are represented by common logarithm of the ratio of upper bound and lower bound of 95% credible intervals. Blue bars represent the case with “B”. Red bars represent the case with “BI”. Green bars represent the case with “BIT_s_”. Magenta bars represent the case with “BIT_e_”. Cyan bars represent the case with “BIT_s_T_e_”.
(PDF)

Figure S15 **Calculated histograms of switching time of caspase-3 activation.** Histogram of switching time of caspase-3 activation calculated with “T_s_”. Red outline box bars represent the approximated histogram of the function shown in Figure 5.E.
(PDF)

Table S1 **Initial concentrations of proteins in dynamics calculation.**
(DOC)

Table S2 **Correlation coefficients between two inferred parameters.**
(DOC)

Text S1 **MCMC-HFM algorithm satisfies detailed balance condition.**
(DOC)

Text S2 **Correlation between inferred parameters for the apoptosis model.**
(DOC)
