# Correction: Bayesian Parameter Inference by Markov Chain Monte Carlo with Hybrid Fitness Measures: Theory and Test in Apoptosis Signal Transduction Network

**DOI:** 10.1371/journal.pone.0097961

**Published:** 2014-05-12

**Authors:** The PLOS ONE Staff

## Notice of Republication

This article was republished on April 21st, 2014, to correct changes to the text, Figure 1, and Table S1. The publisher apologizes for this error. Please download the PDF and Supporting Information again to view the corrected article and supporting table.
