# Supplementary figures and images for: Bayesian Parameter Inference by Markov Chain Monte Carlo with Hybrid Fitness Measures: Theory and Test in Apoptosis Signal Transduction Network
Source: PLoS One. 2013 Sep 27;8(9):e74178. doi: 10.1371/journal.pone.0074178 (PMC3785499; doi:10.1371/journal.pone.0074178)

A

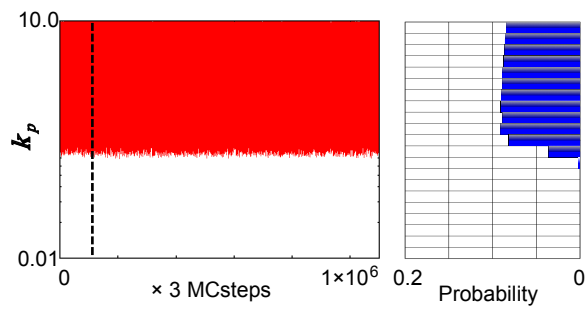

B

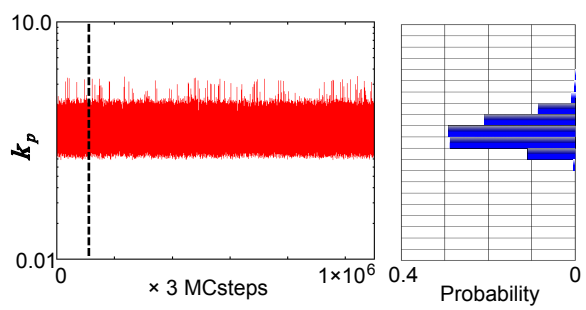

Supplement: Figure S1 — Examples of trace plots of MCMC and probability distributions in the kinetic toy model. (A) Trace plot and the probability distribution of kp using “BI”. (B) Trace plot and the probability distribution of kp using “BITe[Y]time = 100”. Dotted lines indicate the 300000th step, of which left side is the burn in period. (PDF) [file pone.0074178.s001.pdf]

A-a

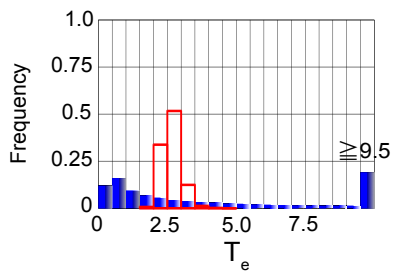

A-b

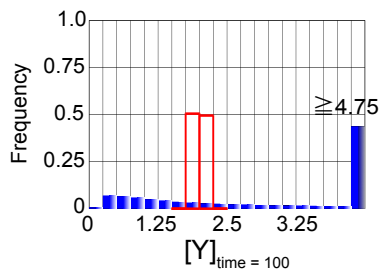

B-a

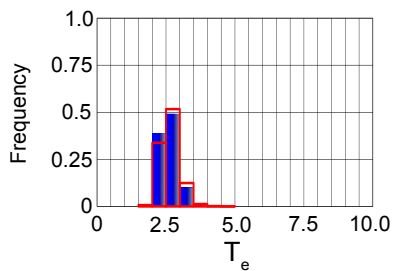

B-b

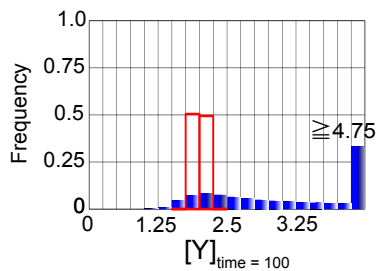

C-a

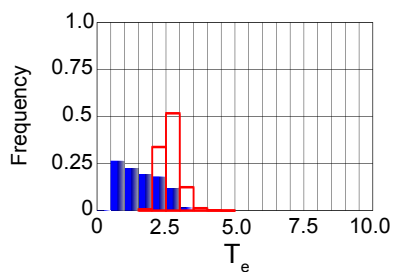

C-b

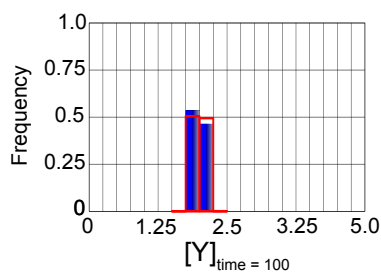

D-a

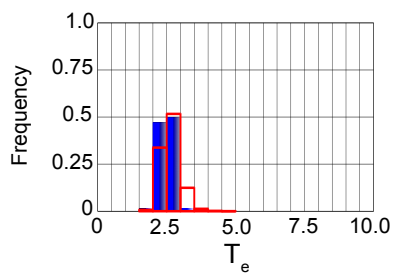

D-b

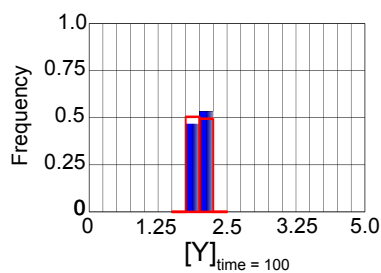

E-a

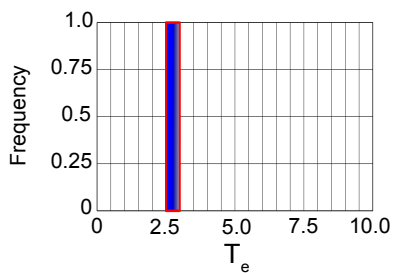

E-b

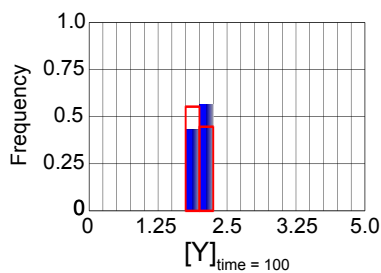

Supplement: Figure S2 — Calculated histograms of execution time of Y production “Te” and concentration of Y at time = 100 “[Y]time = 100”. Histograms of execution time of Y production, “Te”, and concentration of Y at time = 100, “[Y]time = 100” calculated with “BI” (A-a) and (A-b) respectively, those with “BITe” (B-a) and (B-b) respectively, those with “BI[Y]time = 100” (C-a) and (C-b) respectively, those with “BITe[Y]time = 100” (D-a) and (D-b) respectively, and those with “BITe[Y]time = 100” with weaker noise in quantitative fitness (E-a) and (E-b). Blue bars represent calculated results. Red outline box bars represent the histogram generated by adding Gaussian noise into the model. (PDF) [file pone.0074178.s002.pdf]

A

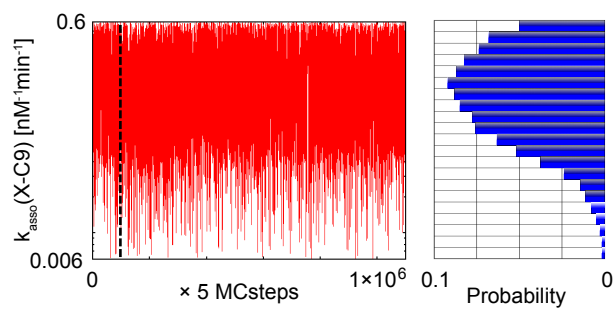

B

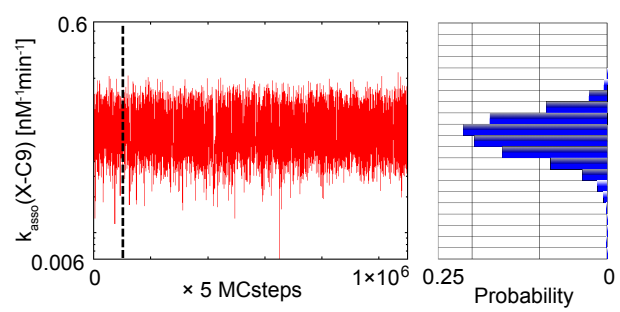

Supplement: Figure S3 — Examples of trace plots of MCMC and probability distributions in the apoptosis model. (A) Trace plot and the probability distribution of kasso (X-C9) with “BI”. (B) Trace plot and the probability distribution of kasso (X-C9) with “BITsTe”. Dotted lines indicate the 500000th step, of which left side is the burn in period. (PDF) [file pone.0074178.s003.pdf]

A

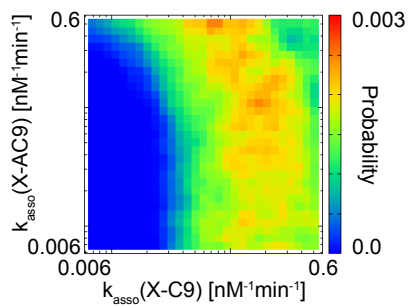

B

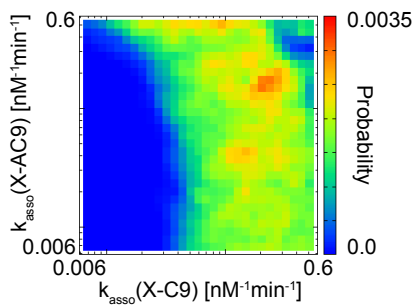

C

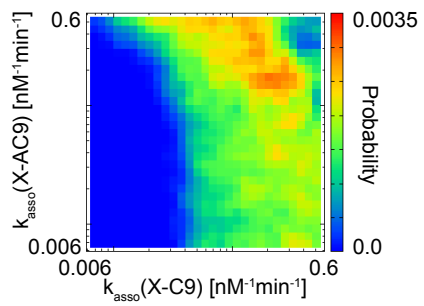

D

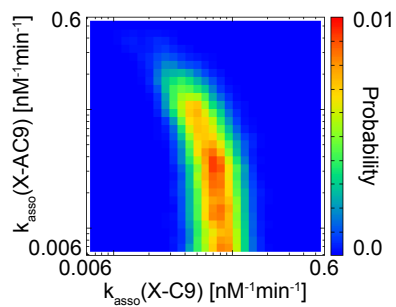

E

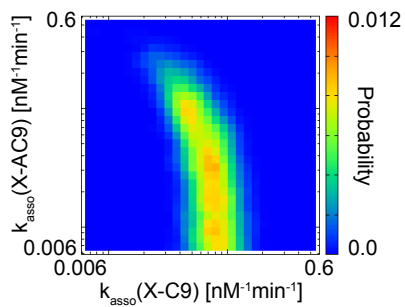

Supplement: Figure S4 — Joint probability distributions of the pair of kasso (X-C9) and kasso (X-AC9). Probability distribution with “B” (A), that with “BI” (B), that with “BITs” (C), that with “BITe” (D), that with “BITsTe” (E). (PDF) [file pone.0074178.s004.pdf]

A

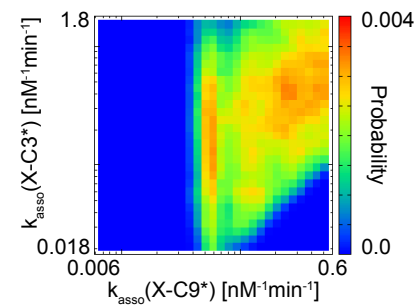

B

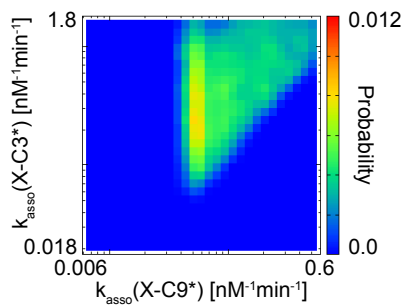

C

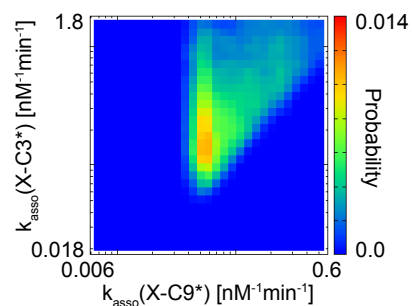

D

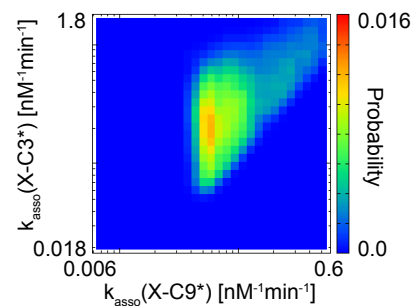

E

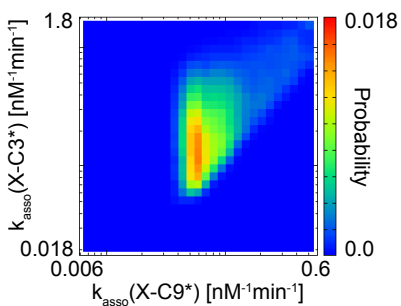

Supplement: Figure S5 — Joint probability distributions of the pair of kasso (X-C9*) and kasso (X-C3*). Probability distribution with “B” (A), that with “BI” (B), that with “BITs” (C), that with “BITe” (D), that with “BITsTe” (E). (PDF) [file pone.0074178.s005.pdf]

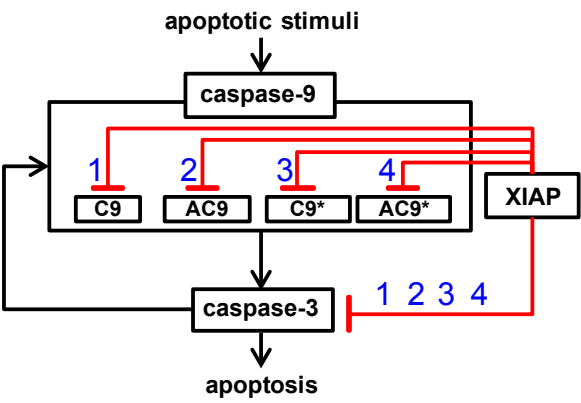

Supplement: Figure S6 — Simplified diagram of apoptosis signal transduction network focused on 4 implicit positive feedbacks. Blue and numbered interactions represent implicit positive feedbacks. 1: C9-X-C3* implicit positive feedback, 2: AC9-X-C3* implicit positive feedback, 3: C9*-X-C3* implicit positive feedback, 4: AC9*-X-C3* implicit positive feedback. (PDF) [file pone.0074178.s006.pdf]

A-a

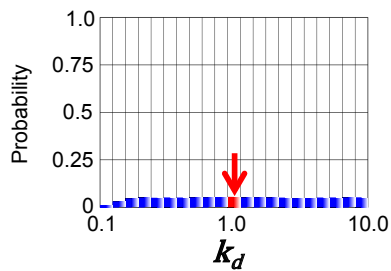

A-b

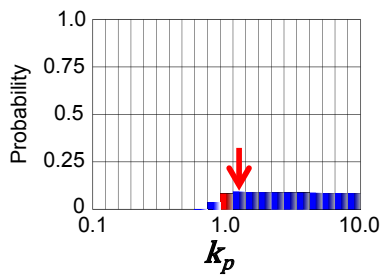

A-c

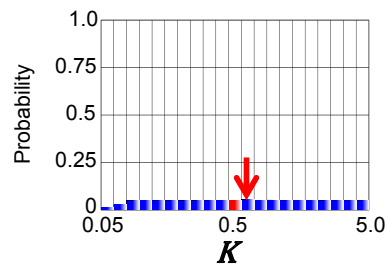

B-a

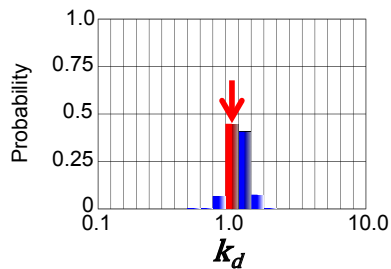

B-b

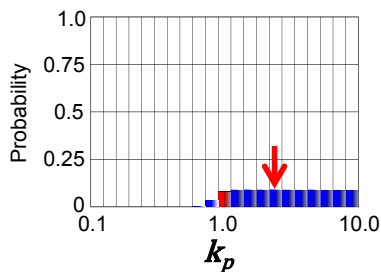

B-c

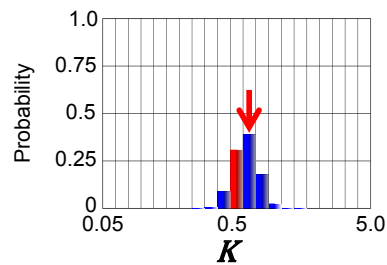

C-a

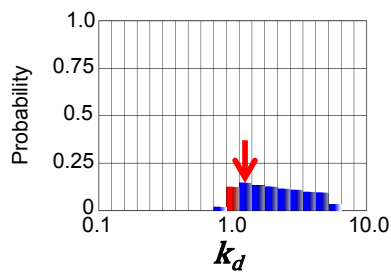

C-b

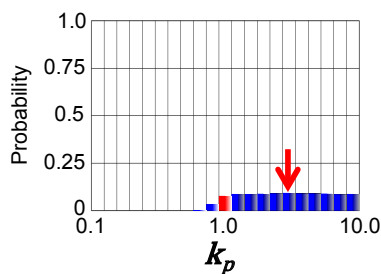

C-c

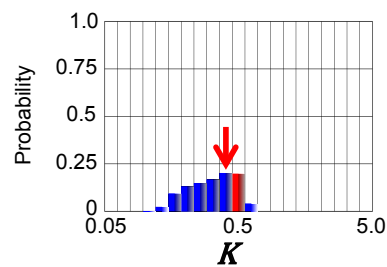

D-a

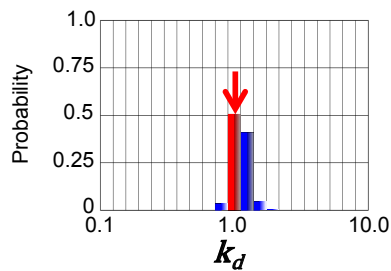

D-b

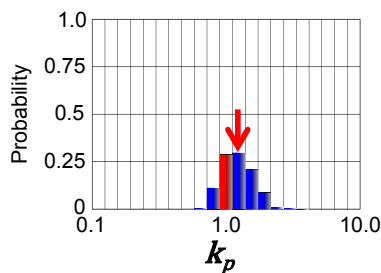

D-c

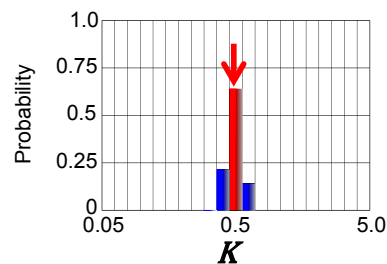

E-a

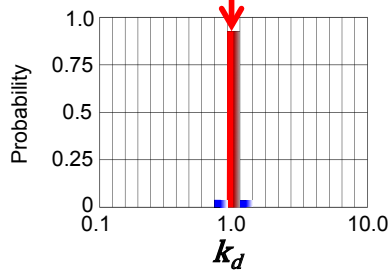

E-b

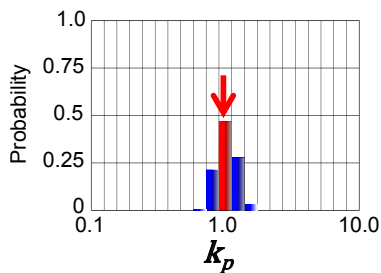

E-c

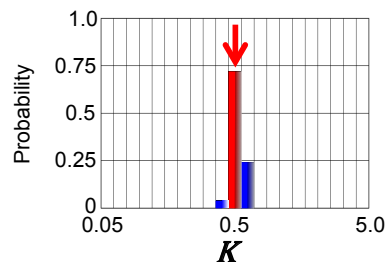

Supplement: Figure S7 — Marginal probability distributions of parameters in the kinetic toy model (proposal distribution in MCMC set to normal distribution). Probability distributions with “BI” (A), those with “BITe” (B), those with “BI[Y]time = 100” (C), those with “BITe[Y]time = 100” (D), and those with ”BITe[Y]time = 100” with weaker noise in quantitative fitness (see main text) (E). Red bars represent the “true” values of parameters. Red arrows indicate the modes. (PDF) [file pone.0074178.s007.pdf]

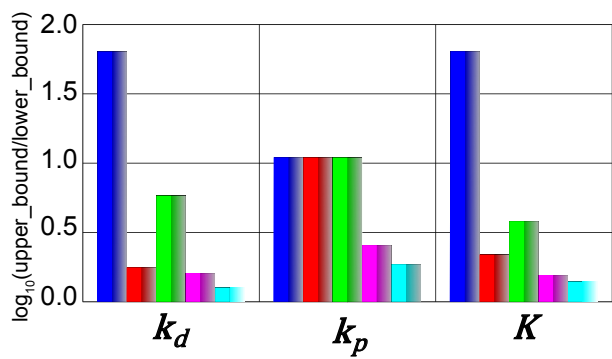

Supplement: Figure S8 — 95% credible intervals of inferred parameters in the kinetic toy model (proposal distribution in MCMC set to normal distribution). The 95% credible intervals are represented by common logarithm of the ratio of upper bound and lower bound of 95% credible intervals. Blue bars represent the case with “BI”. Red bars represent the case with “BITe”. Green bars represent the case with “BI[Y]time = 100”. Magenta bars represent the case with “BITe [Y]time = 100”. Cyan bars represent the case with “BITe [Y]time = 100” with weaker noise in quantitative fitness. (PDF) [file pone.0074178.s008.pdf]

A

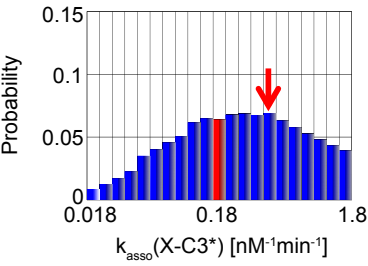

B

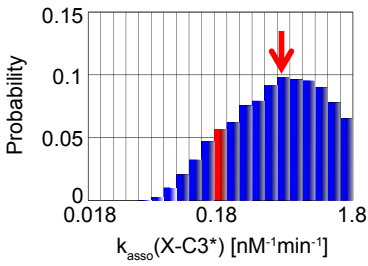

C

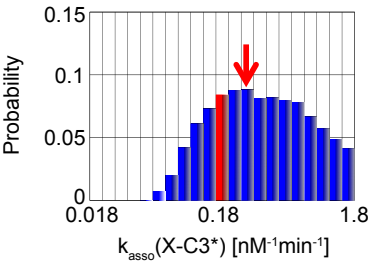

D

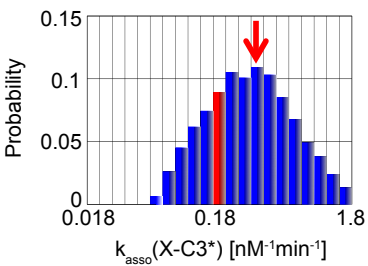

E

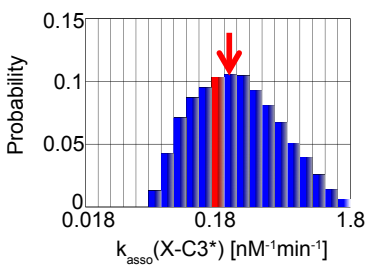

Supplement: Figure S9 — Marginal probability distributions of kasso (X-C3*) (proposal distribution in MCMC set to normal distribution). Probability distribution with “B” (A), that with “BI” (B), that with “BITs” (C), that with “BITe” (D), and that with ”BITsTe” (E). Red bars represent experimentally estimated values. Red arrows indicate the modes. (PDF) [file pone.0074178.s009.pdf]

A

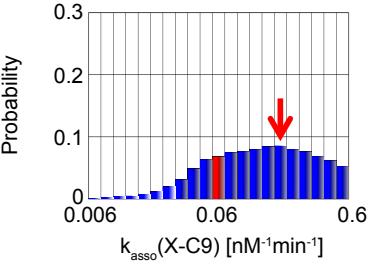

B

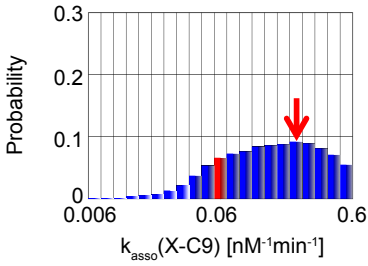

C

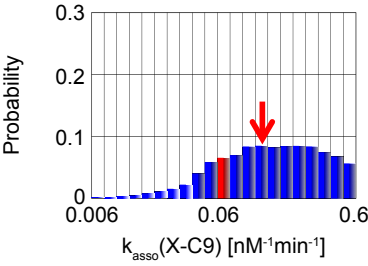

D

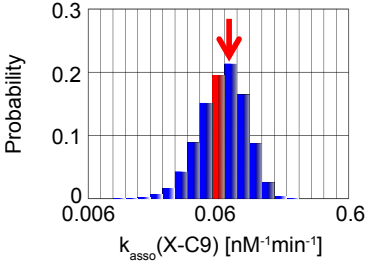

E

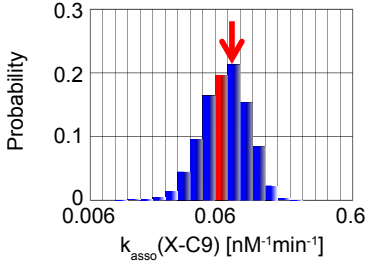

Supplement: Figure S10 — Marginal probability distributions of kasso (X-C9) (proposal distribution in MCMC set to normal distribution). Probability distribution with “B” (A), that with “BI” (B), that with “BITs” (C), that with “BITe” (D), and that with ”BITsTe” (E). Red bars represent experimentally estimated values. Red arrows indicate the modes. (PDF) [file pone.0074178.s010.pdf]

A

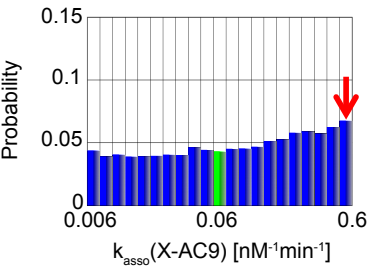

B

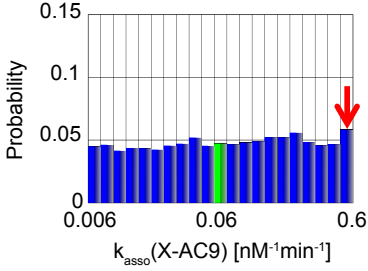

C

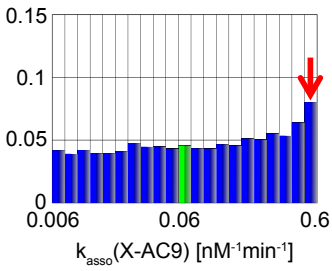

D

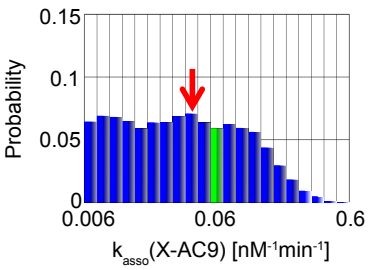

E

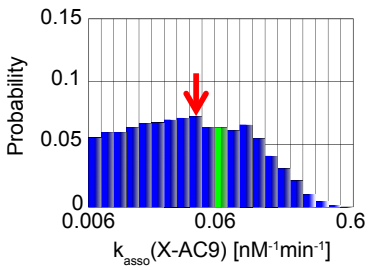

Supplement: Figure S11 — Marginal probability distributions of kasso (X-AC9) (proposal distribution in MCMC set to normal distribution). Probability distribution with “B” (A), that with “BI” (B), that with “BITs” (C), that with “BITe” (D), and that with ”BITsTe” (E). Green bars represent the used value in Legewie et al's study but not experimentally estimated. Red arrows indicate the modes. (PDF) [file pone.0074178.s011.pdf]

A

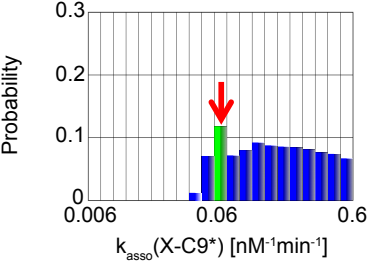

B

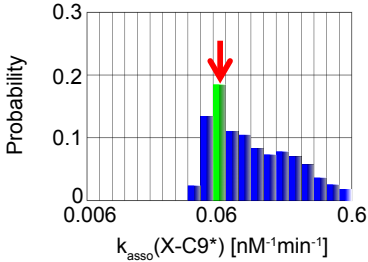

C

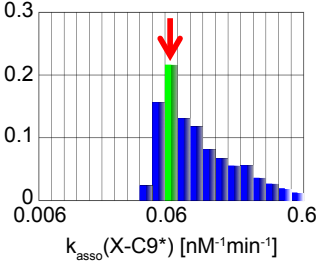

D

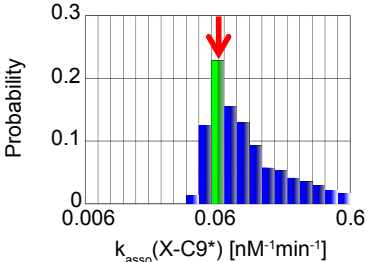

E

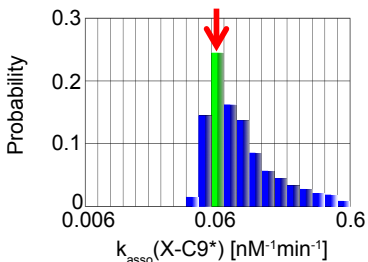

Supplement: Figure S12 — Marginal probability distributions of kasso (X-C9*) (proposal distribution in MCMC set to normal distribution). Probability distribution with “B” (A), that with “BI” (B), that with “BITs” (C), that with “BITe” (D), and that with ”BITsTe” (E). Green bars represent the used value in Legewie et al's study but not experimentally estimated. Red arrows indicate the modes. (PDF) [file pone.0074178.s012.pdf]

A

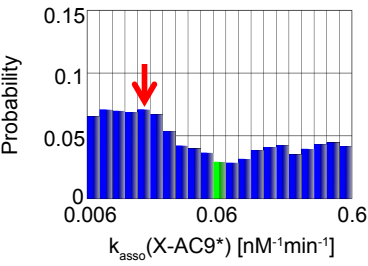

B

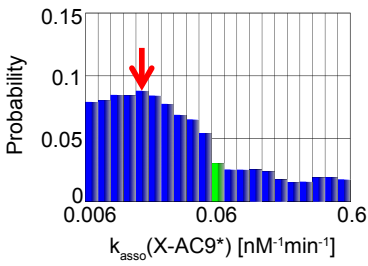

C

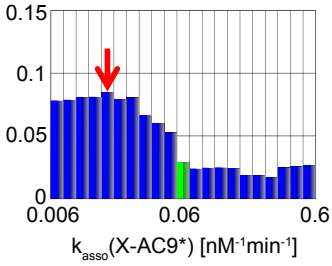

D

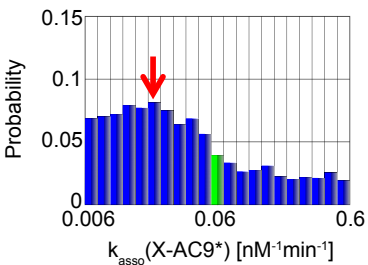

E

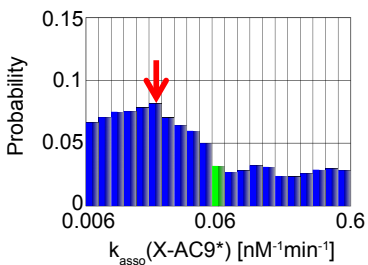

Supplement: Figure S13 — Marginal probability distributions of kasso (X-AC9*) (proposal distribution in MCMC set to normal distribution). Probability distribution with “B” (A), that with “BI” (B), that with “BITs” (C), that with “BITe” (D), and that with ”BITsTe” (E). Green bars represent the used value in Legewie et al's study but not experimentally estimated. Red arrows indicate the modes. (PDF) [file pone.0074178.s013.pdf]

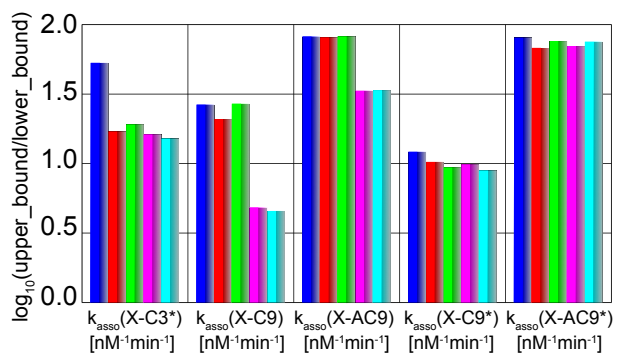

Supplement: Figure S14 — 95% credible intervals of inferred parameters in the apoptosis model (proposal distribution in MCMC set to normal distribution). The 95% credible intervals are represented by common logarithm of the ratio of upper bound and lower bound of 95% credible intervals. Blue bars represent the case with “B”. Red bars represent the case with “BI”. Green bars represent the case with “BITs”. Magenta bars represent the case with “BITe”. Cyan bars represent the case with “BITsTe”. (PDF) [file pone.0074178.s014.pdf]

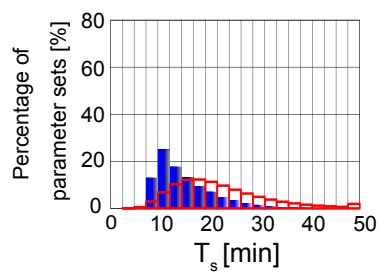

Supplement: Figure S15 — Calculated histograms of switching time of caspase-3 activation. Histogram of switching time of caspase-3 activation calculated with “Ts”. Red outline box bars represent the approximated histogram of the function shown in Figure 5.E. (PDF) [file pone.0074178.s015.pdf]
