# Supplementary material for: Bayesian Parameter Inference by Markov Chain Monte Carlo with Hybrid Fitness Measures: Theory and Test in Apoptosis Signal Transduction Network
Source: PLoS One. 2013 Sep 27;8(9):e74178. doi: 10.1371/journal.pone.0074178 (PMC3785499; doi:10.1371/journal.pone.0074178)
Supplement: Table S1 — Initial concentrations of proteins in dynamics calculation. (DOC) [file pone.0074178.s016.doc]

**Table S1. Initial concentrations of proteins in dynamics calculation.**

| Protein | Initial concentration [nM] |
| --- | --- |
| A | 20 |
| C9 | 20 |
| C9* | 0 |
| AC9 | 0 |
| AC9* | 0 |
| C3 | 200 |
| C3* | 0 |
| X | 40 |
| C9X | 0 |
| C9*X | 0 |
| AC9X | 0 |
| AC9*X | 0 |
| C3*X | 0 |

Abbreviations are as follows: A: Apaf-1, C9: caspase-9, C3: caspse-3, X: XIAP.
