# Supplementary material for: Bayesian Parameter Inference by Markov Chain Monte Carlo with Hybrid Fitness Measures: Theory and Test in Apoptosis Signal Transduction Network
Source: PLoS One. 2013 Sep 27;8(9):e74178. doi: 10.1371/journal.pone.0074178 (PMC3785499; doi:10.1371/journal.pone.0074178)
Supplement: Table S2 — Correlation coefficients between two inferred parameters. (DOC) [file pone.0074178.s017.doc]

**TableS2. Correlation coefficients between two inferred parameters.**

|  | B | BI | BIT_s_ | BIT_e_ | BIT_s_T_e_ |
| --- | --- | --- | --- | --- | --- |
| k_asso_ (X-C9) / k_asso_ (X-AC9) | -0.251 | -0.321 | -0.328 | -0.557 | -0.572 |
| k_asso_ (X-C9) / k_asso_ (X-C9*) | -0.202 | -0.092 | -0.071 | -0.168 | -0.178 |
| k_asso_ (X-C9) / k_asso_ (X-AC9*) | -0.065 | -0.009 | 0.019 | -0.098 | -0.101 |
| k_asso_ (X-C9) / k_asso_ (X-C3*) | 0.000 | 0.356 | 0.328 | 0.185 | 0.131 |
| k_asso_ (X-AC9) / k_asso_ (X-C9*) | -0.024 | 0.050 | 0.085 | -0.014 | 0.043 |
| k_asso_ (X-AC9) / k_asso_ (X-AC9*) | 0.016 | 0.022 | 0.019 | -0.042 | -0.050 |
| k_asso_ (X-AC9) / k_asso_ (X-C3*) | -0.087 | -0.084 | -0.060 | -0.152 | -0.094 |
| k_asso_ (X-C9*) / k_asso_ (X-AC9*) | 0.031 | 0.109 | 0.092 | 0.221 | 0.188 |
| k_asso_ (X-C9*) / k_asso_ (X-C3*) | 0.305 | 0.460 | 0.509 | 0.622 | 0.629 |
| k_asso_ (X-AC9*) / k_asso_ (X-C3*) | 0.002 | 0.101 | 0.085 | 0.192 | 0.145 |
