# Supplementary material for: Bayesian Parameter Inference by Markov Chain Monte Carlo with Hybrid Fitness Measures: Theory and Test in Apoptosis Signal Transduction Network
Source: PLoS One. 2013 Sep 27;8(9):e74178. doi: 10.1371/journal.pone.0074178 (PMC3785499; doi:10.1371/journal.pone.0074178)
Supplement: Text S1 — MCMC-HFM algorithm satisfies detailed balance condition. (DOC) [file pone.0074178.s018.doc]

**Text S1.MCMC-HFM algorithm satisfies detailed balance condition.**

MCMC-HFM algorithm satisfies detail balance condition as shown below.

*Proof*:

Denote the transition mechanism of the chain by.

We consider the case

$$\frac{\text{ f}_{\text{quant}}\left(\text{z}_{\text{n}}\left(\text{θ'}\right)\right)\text{ ... f}_{\text{quant}}\left(\text{z}_{\text{m}+1}\left(\text{θ'}\right)\right)\text{I}\left(\text{C}_{\text{m}}\left(\text{θ'}\right)\right)\text{ ... I}\left(\text{C}_{1}\left(\text{θ'}\right)\right)\text{ π}\left(\text{θ'}\right)\text{ q}\left(\text{θ|θ'}\right)\text{ }}{\text{ f}_{\text{quant}}\left(\text{z}_{\text{n}}\left(\text{θ}\right)\right)\text{ ... f}_{\text{quant}}\left(\text{z}_{\text{m}+1}\left(\text{θ}\right)\right)\text{I}\left(\text{C}_{\text{m}}\left(\text{θ}\right)\right)\text{ ... I}\left(\text{C}_{1}\left(\text{θ}\right)\right)\text{ π}\left(\text{θ}\right)\text{ q}\left(\text{θ'|θ}\right)}\leq\text{ 1 ... Eq}$$

Then,

$$\text{ f}_{\text{quant}}\left(\text{z}_{\text{n}}\left(\text{θ}\right)\right)\text{ ... f}_{\text{quant}}\left(\text{z}_{\text{m}+1}\left(\text{θ}\right)\right)\text{I}\left(\text{C}_{\text{m}}\left(\text{θ}\right)\right)\text{ ... I}\left(\text{C}_{1}\left(\text{θ}\right)\right)\text{ π}\left(\text{θ}\right)\text{ T }\left(\text{θ}\to\text{θ}'\right)()$$

$$\text{ }=\text{f}_{\text{quant}}\left(\text{z}_{\text{n}}\left(\text{θ}\right)\right)\text{ ... f}_{\text{quant}}\left(\text{z}_{\text{m}+1}\left(\text{θ}\right)\right)\text{I}\left(\text{C}_{\text{m}}\left(\text{θ}\right)\right)\text{ ... I}\left(\text{C}_{1}\left(\text{θ}\right)\right)\text{ π}\left(\text{θ}\right)\text{ q}\left(\text{θ'|θ}\right)\text{α}\left(\text{θ,}\text{θ}'\right)()$$

$$\text{ }=\text{f}_{\text{quant}}\left(\text{z}_{\text{n}}\left(\text{θ}\right)\right)\text{ ... f}_{\text{quant}}\left(\text{z}_{\text{m}+1}\left(\text{θ}\right)\right)\text{I}\left(\text{C}_{\text{m}}\left(\text{θ}\right)\right)\text{ ... I}\left(\text{C}_{1}\left(\text{θ}\right)\right)\text{ π}\left(\text{θ}\right)\text{ q}\left(\text{θ'|θ}\right)$$

$$\text{ }\times\frac{\text{ f}_{\text{quant}}\left(\text{z}_{\text{n}}\left(\text{θ'}\right)\right)\text{ ... f}_{\text{quant}}\left(\text{z}_{\text{m}+1}\left(\text{θ'}\right)\right)\text{I}\left(\text{C}_{\text{m}}\left(\text{θ'}\right)\right)\text{ ... I}\left(\text{C}_{1}\left(\text{θ'}\right)\right)\text{ π}\left(\text{θ'}\right)\text{ q}\left(\text{θ|θ'}\right)\text{ }}{\text{ f}_{\text{quant}}\left(\text{z}_{\text{n}}\left(\text{θ}\right)\right)\text{ ... f}_{\text{quant}}\left(\text{z}_{\text{m}+1}\left(\text{θ}\right)\right)\text{I}\left(\text{C}_{\text{m}}\left(\text{θ}\right)\right)\text{ ... I}\left(\text{C}_{1}\left(\text{θ}\right)\right)\text{ π}\left(\text{θ}\right)\text{ q}\left(\text{θ'|θ}\right)}$$

$$\text{ }=\text{f}_{\text{quant}}\left(\text{z}_{\text{n}}\left(\text{θ'}\right)\right)\text{ ... f}_{\text{quant}}\left(\text{z}_{\text{m}+1}\left(\text{θ'}\right)\right)\text{I}\left(\text{C}_{\text{m}}\left(\text{θ'}\right)\right)\text{ ... I}\left(\text{C}_{1}\left(\text{θ'}\right)\right)\text{ π}\left(\text{θ'}\right)\text{ q}\left(\text{θ|θ'}\right)$$

$$\text{ }=\text{f}_{\text{quant}}\left(\text{z}_{\text{n}}\left(\text{θ'}\right)\right)\text{ ... f}_{\text{quant}}\left(\text{z}_{\text{m}+1}\left(\text{θ'}\right)\right)\text{I}\left(\text{C}_{\text{m}}\left(\text{θ'}\right)\right)\text{ ... I}\left(\text{C}_{1}\left(\text{θ'}\right)\right)\text{ π}\left(\text{θ'}\right)\text{ q}\left(\text{θ|θ'}\right)\text{α}\left(\text{θ',θ}\right)\text{ (α}\left(\text{θ',θ}\right)=1\text{)}$$

$$\text{ }=\text{f}_{\text{quant}}\left(\text{z}_{\text{n}}\left(\text{θ'}\right)\right)\text{ ... f}_{\text{quant}}\left(\text{z}_{\text{m}+1}\left(\text{θ'}\right)\right)\text{I}\left(\text{C}_{\text{m}}\left(\text{θ'}\right)\right)\text{ ... I}\left(\text{C}_{1}\left(\text{θ'}\right)\right)\text{ π}\left(\text{θ'}\right)\text{ T}\left(\text{θ'}\to\text{θ}\right)$$

The case the left of Eq > 1 is analogously proved. A posterior distribution satisfies detailed balance condition.
