# Supplementary material for: Bayesian Parameter Inference by Markov Chain Monte Carlo with Hybrid Fitness Measures: Theory and Test in Apoptosis Signal Transduction Network
Source: PLoS One. 2013 Sep 27;8(9):e74178. doi: 10.1371/journal.pone.0074178 (PMC3785499; doi:10.1371/journal.pone.0074178)
Supplement: Text S2 — Correlation between inferred parameters for the apoptosis model. (DOC) [file pone.0074178.s019.doc]

**Text S2. Correlation between inferred parameters for the apoptosis model.**

We examined the correlations between inferred parameters to gain deeper understanding of the implicit positive feedback. Here, we inferred five kinetic parameters and thus there were totally ten pairs of kinetic parameters. Correlation coefficients between two kinetic parameters are shown in Table S2. Of the ten pairs, we focus on the two pairs which showed relatively strong correlation; the pair of k_asso_ (X-C9) and k_asso_ (X-AC9) and the pair of k_asso_ (X-C9*) and k_asso_ (X-C3*). Joint probability distributions of these two pairs are shown in Figure S4 and S5.

The region of joint probability distributions of k_asso_ (X-C9) and k_asso_ (X-AC9) became clearly narrower when additional information “T_e_” was added (Figure S4). The correlation coefficient between k_asso_ (X-C9) and k_asso_ (X-AC9) showed negative correlation and it also became stronger when additional information “T_e_” was added, although still the correlation was not very strong (Table S2). These results indicate the similar content to the credible intervals of inferred parameters in Figure 13. That is, to realize the appropriate timing of casapse-3 activation after MOMP, 10~20 minutes, amounts of free C9 and free AC9 need to exist in well-balanced, since C9 and AC9 determine the switch-on timing of the positive feedbacks and thus determine the execution time of caspase-3 activation. The negative correlation coefficients seem to reflect the necessary balance for the existence of free C9 amount and free AC9 amount. The narrower regions of joint probability distributions and stronger correlation coefficients seem to reflect the stronger constraints for execution time of caspase-3 activation.

The region of joint probability distributions of k_asso_ (X-C9*) and k_asso_ (X-C3*) became narrower and the positive correlation slightly increases from R = 0.305 to R = 0.460 when the qualitative condition on the existence of “I” was added (Figure S5, Table S2). Although correlation coefficient is not very large, these results might be interpreted as follows. As explained in former sections, to realize irreversibility of caspase-3 activation, k_asso_ (X-C3*) needs to favor relatively larger values (Figure 6). Then, this tendency of k_asso_ (X-C3*) would influence k_asso_ (X-C9*). This seems to suggest that, for the irreversibility of caspase-3 activation, the implicit positive feedback C9*-X-C3* was more important than other three implicit positive feedbacks, C9-X-C3*, AC9-X-C3* and AC9*-X-C3* (Figure S6). Compared to the addition of information of irreversibility, the addition of information of switching time of caspase-3 activation, “T_s_”, did not apparently change the acceptable region and correlation coefficients very strongly (Figure S5, Table S2). Although additional information “T_e_” also did not apparently change the acceptable region very strongly (Figure S5), the correlation coefficient increased (Table S2). This might be explained as follows. The larger correlation coefficient seems to indirectly reflect the constraint to the balance between free C9 and free AC9. As illustrated above, amounts of free C9 and free AC9 need to exist in well-balanced to realize the appropriate timing of caspase-3 activation, 10~20 minutes after MOMP. This constraint to k_asso_ (X-C9) and k_asso_ (X-AC9) indirectly influence the C9*-X-C3* positive feedback because both C9 and AC9 also form C9-X-C3* positive feedback and AC9-X-C3* positive feedback (Figure S6). Thus, the case with “T_e_” has larger correlation coefficient than the case without the fitness to realize appropriate timing of caspase-3 activation indirectly.

Taken together, the analysis of joint probability distributions and correlations suggests that free C9 and free AC9 amounts are especially important to determine the timing of caspase-3 activation after MOMP i.e. execution time of caspase-3 activation. The analysis also suggests that C9*-X-C3* implicit positive feedback is more important than other three implicit positive feedbacks for irreversibility of caspase-3 activation.

In this manner, we could specify and understand the important relationships between kinetic parameters and corresponding biochemical processes for irreversibility and timing of caspase-3 activation after MOMP with parameter sets generated by MCMC-HFM algorithm.
